# Toward a unified taxonomy of information dynamics via Integrated Information Decomposition

**DOI:** 10.1073/pnas.2423297122

**Published:** 2025-09-22

**Authors:** Pedro A. M. Mediano, Fernando E. Rosas, Andrea I. Luppi, Robin L. Carhart-Harris, Daniel Bor, Anil K. Seth, Adam B. Barrett

**Affiliations:** ^a^Department of Computing, Imperial College London, London SW7 2AZ, United Kingdom; ^b^Division of Psychology and Language Sciences, University College London, London WC1H 0AP, United Kingdom; ^c^Department of Informatics, Sussex AI and Sussex Centre for Consciousness Science, University of Sussex, Brighton BN1 9QJ, United Kingdom; ^d^Centre for Complexity Science, Department of Mathematics, and Centre for Psychedelic Research, Department of Brain Sciences, Imperial College London, London SW7 2AZ, United Kingdom; ^e^Principles of Intelligent Behavior in Biological and Social Systems, Prague 182 00, Czech Republic; ^f^Department of Psychiatry, Centre for Eudaimonia and Human Flourishing, University of Oxford, Oxford OX3 7JX, United Kingdom; ^g^St. John’s College, University of Cambridge, Cambridge CB2 1TP, United Kingdom; ^h^Division of Information Engineering, University of Cambridge, Cambridge CB2 1PZ, United Kingdom; ^i^Department of Neurology, University of California San Francisco, San Francisco, CA 94158; ^j^Department of Psychology, Queen Mary University of London, London E1 4NS, United Kingdom; ^k^Department of Psychology, University of Cambridge, Cambridge CB2 3EB, United Kingdom; ^l^Program on Brain, Mind, and Consciousness, Canadian Institute for Advanced Research, Toronto, Ontario M5G 1M1, Canada; ^m^The Data Intensive Science Centre, University of Sussex, Brighton BN1 9QJ, United Kingdom

**Keywords:** information theory, complexity, dynamical systems, integrated information

## Abstract

Our ability to understand and control complex systems of many interacting parts remains limited. A key challenge is that we still do not know how best to describe—and quantify—the many-to-many dynamical interactions that characterize their complexity. To address this limitation, we introduce the mathematical framework of Integrated Information Decomposition, or ΦID. ΦID provides a comprehensive framework to disentangle and characterize the information dynamics of complex multivariate systems. On the theoretical side, ΦID reveals the existence of previously unreported modes of collective information flow, providing tools to express well-known measures of information transfer, information storage, and dynamical complexity as aggregates of these modes, thereby overcoming some of their known theoretical shortcomings. On the empirical side, we validate our theoretical results with computational models and examples from over 1,000 biological, social, physical, and synthetic dynamical systems. Altogether, ΦID improves our understanding of the behavior of widely used measures for characterizing complex systems across disciplines and leads to new more refined analyses of dynamical complexity.

## Significance

Complex systems, from the human brain to the global economy, are made of multiple elements that interact dynamically, often giving rise to collective behaviors that are not readily predictable from the “sum of the parts.” To advance our understanding of how this can occur, here we present a mathematical framework to disentangle and quantify different “modes” of information storage, transfer, and integration in complex systems. This framework reveals previously unreported collective behavior phenomena in experimental data across scientific fields, and provides principles to classify and formally relate diverse measures of dynamical complexity and information processing.

How can we best characterize the plethora of dynamical phenomena that can emerge in a system of interacting components? Progress on this question seems critical to support advances in our ability to understand, predict, and control complex systems such as the central nervous system (1), the global climate (2), macroeconomics (3), and many others.

Various one-dimensional metrics have been proposed to assess the dynamical complexity of processes (e.g. refs. 4 and 5). An interesting case of this is found in the neuroscience literature, where it has been proposed that a key feature of the neural dynamics underpinning advanced cognition, flexible behavior, and even the presence of consciousness, can be captured by a single number that accounts for the brain’s ability to “integrate information.” There have been several attempts to operationalize this notion, including the various Φ measures in integrated information theory (IIT) (6–9) and causal density (CD) (10). However, these measures have been shown to behave inconsistently (11–13), making empirical applications difficult to interpret.

In addition to one-dimensional indicators of overall dynamical complexity, alternative approaches such as transfer entropy (14, 15) provide complementary insights by quantifying specific types of interactions between parts of the system, such as the strength and direction of information flow or information storage. However, in recent years it has become apparent that we are missing a conceptual framework for linking indicators of global dynamical complexity, and measures of part–part interactions. For example, what is the relationship of information transfer with information storage, or with the various proposed measures of dynamical complexity? Although individual relationships have been identified [for example, between transfer entropy and causal density (10) or geometric integrated information (16)], a unifying mathematical account that brings together each of these different dynamical phenomena remains missing.

A promising formalism to account for such diversity is the framework of Partial Information Decomposition (PID), which demonstrates how the information that two or more sources provide about a given target can be decomposed into redundant, unique, and synergistic components (17). Specifically, redundancy refers to information provided simultaneously by both sources, unique information is information provided by one source but not the other, and synergy is the information conveyed by both sources together but none of them in isolation. In their seminal work, Williams and Beer demonstrated how well-known measures of information dynamics—such as transfer entropy and active information storage (18)—are themselves not monolithic, but rather they are aggregates of these fundamental “information atoms” (17). Earlier approaches had already recognized the difference between synergy and redundancy (19–26), but a single “synergy-redundancy” balance index can be difficult to interpret when synergy and redundancy coexist(17, 27, 28). By quantifying each information atom separately, PID overcomes this limitation—though at the cost of introducing challenges related to operationalization and scalability. Despite these challenges, PID has found successful applications across a variety of systems, from cellular automata (29, 30), artificial neural networks (31–33), to socioeconomic data (34), gene interactions (35), and neuroscience (28). However, the PID formalism suffers from a fundamental limitation: The taxonomy of information phenomena introduced by PID is only valid in scenarios with many sources of information but only a single target variable. This prevents PID from providing a full picture of the dynamics of complex systems, where many-to-many interactions are ubiquitous.

Here, we address this critical limitation by introducing a more extensive taxonomy of information dynamics through the *Integrated Information Decomposition* framework (ΦID). This framework reveals how widely used measures of information integration, transfer, and storage are aggregates of several qualitatively distinct types of information dynamics. As proof of concept, we analyze 1,053 datasets of multivariate time-series from a wide variety of biological, physical, social, and artificial systems (36), demonstrating that the information-dynamic phenomena identified by ΦID can have substantial empirical consequences for real-world analyses and their interpretation.

## Integrated Information Decomposition: ΦID

### Decomposing Multivariate Information.

Consider a system composed of two interdependent elements that coevolve over time. If the system’s future state depends only on the preceding state (i.e. if its dynamics are Markovian), then the total amount of information carried from past to future is known as the time-delayed mutual information^*^ (12), and can be quantified as the mutual information between past and future states of the system:

[1] $$\begin{array}{c} \text{TDMI}=I\left(X_{t};X_{t+1}\right)=I\left(X_{t}^{1},X_{t}^{2}\;;\;X_{t+1}^{1},X_{t+1}^{2}\right)\;. \end{array}$$

Above, the superscripts 1 and 2 refer to the two elements of the system, and $X_{t}=\left(X_{t}^{1},X_{t}^{2}\right)$ is a shorthand notation for the system’s state at time t.

One can analyze the total information flow using the PID framework, which decomposes the mutual information between multiple sources and a target variable into unique (Un), redundant (Red), and synergistic (Syn) contributions—also known as “information atoms” (17). However, just as the strength of PID is its capacity to account for multiple sources of information, one of its main limitations is that it is restricted to considering only a single (potentially multivariate) target. Therefore, a direct application of PID to the TDMI would have to consider the past states of the system’s elements $X_{t}^{1}$ and $X_{t}^{2}$ as sources and the joint future state of the system $X_{t+1}$ as target. Specifically, focusing on how information flows from past to future, this account decomposes the information provided by past states $X_{t}^{1}$ and $X_{t}^{2}$ about the joint future state $X_{t+1}$, as

$$\begin{array}{cc} \text{TDMI} & =\mathrm{Red}\left(X_{t}^{1},X_{t}^{2};X_{t+1}\right)+\mathrm{Un}\left(X_{t}^{1};X_{t+1}|X_{t}^{2}\right)\\ & +\mathrm{Un}\left(X_{t}^{2};X_{t+1}|X_{t}^{1}\right)+\mathrm{Syn}\left(X_{t}^{1},X_{t}^{2};X_{t+1}\right). \end{array}$$

Here, the first term corresponds to the redundant information provided by both $X_{t}^{1}$ and $X_{t}^{2}$ about the joint future state of the system $X_{t+1}$; the second and third terms refer to the unique information that only the past state $X_{t}^{1}$ provides about the joint future state $X_{t+1}$ (and likewise for $X_{t}^{2}$); and finally, the last term accounts for the synergistic information that the two elements’ past states provide about the system’s joint future, only when they are considered together. Unfortunately, this approach neglects the fact that the parts of the system are distinct not only in the past but also in the future—in other words, it can tell where the information is coming from, but not where it is going to. One partial solution would be to consider the time-reverse of the equation above, with both future states as sources and the joint past state as target. However, this leaves unsolved the underlying problem that PID cannot provide a unified decomposition of information across multiple sources and multiple targets simultaneously.

In order to obtain an encompassing description of the system’s dynamics, one must extend the PID approach to enable multitarget information decomposition. To address this issue, our strategy is to take a temporal perspective on PID itself, focusing on how the information encoded by the PID atoms may evolve over time. For instance, information that was uniquely encoded by one element of the system in the past may become redundantly encoded by two in the future, or synergistic information may subsequently become uniquely encoded by one of the elements—and so on. This intuition suggests that when decomposing information flow between past and future in a system of two elements, there are not four, but rather 16 distinct information atoms: each corresponding to a pair of the original four PID atoms evolving from past to future. The mathematical framework that describes this set of 16 information atoms and the relationships between them constitute our key contribution, Integrated Information Decomposition (ΦID). Accordingly, we denote each ΦID atom as a pair of PID atoms: e.g., the information that was carried redundantly in the past and becomes synergistic in the future corresponds to Red→Syn; and the synergistic information in the past that becomes unique to $X_{t+1}^{1}$ in the future corresponds to $\text{Syn}\to {\text{Un}}^{1}$; and so on.

**Fig. 1.**
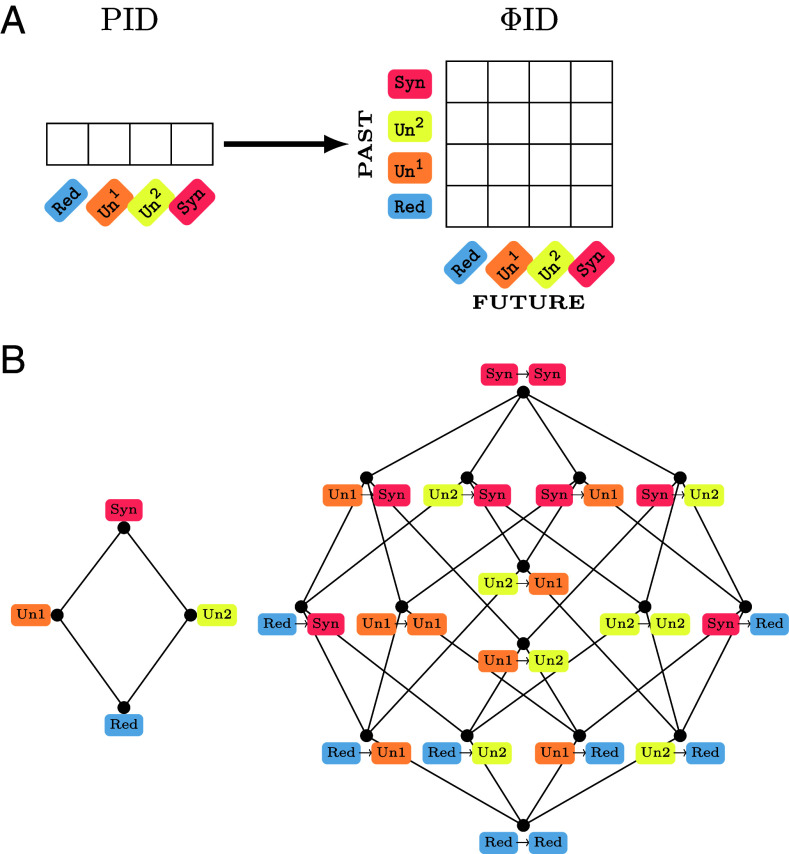
Lattice and matrix representations of information atoms from PID to ΦID. (*A*) In a system of two elements, PID atoms can be represented as an array with four elements. Correspondingly, ΦID atoms can be represented by a matrix, where each atom corresponds to a combination of redundant, unique, or synergistic information in the past and future. (*B*) Representation of PID and ΦID as lattices, where low-order atoms appear below high-order atoms.

Like the original PID atoms, the ΦID atoms are structured in a lattice, depicted in Fig. 1 (for a formal derivation see *Materials and Methods*). As a generalization of PID, ΦID inherits one of its main limitations: the need to specify additional ingredients to calculate the numerical value of these atoms. Just as the PID for two sources yields an underdetermined system of 3 equations for 4 atoms (17), ΦID specifies a system of 15 equations for 16 atoms—and therefore for computing these atoms one must provide one extra constraint. For PID, it is often the redundancy function that provides the needed constraint to allow computation of numerical values for the information atoms. Multiple redundancy functions have been proposed, each satisfying different combinations of desiderata (37–41) (see ref. 42 for a categorization). The same can be done for ΦID by introducing a double-redundancy function—a multitarget extension of PID’s redundancy function. Hence, just like PID admit multiple redundancy functions, so the ΦID framework does not impose a particular functional form for the double-redundancy function, and hence different functional forms can be explored. In the sequel, we adopt the minimum mutual information (MMI) notion of redundancy from PID, which we extend to ΦID. See *Materials and Methods* for formal development of these ideas, and extension to systems of more than two components.

### An Extended Taxonomy for Information Dynamics in Complex Systems.

**Fig. 2.**
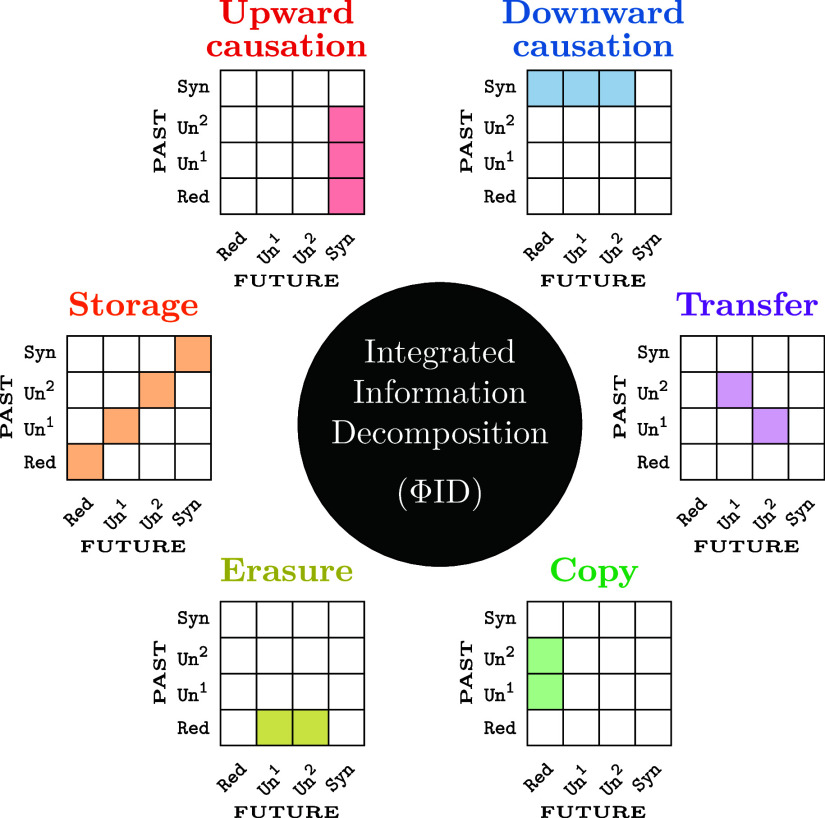
Taxonomy of information dynamics in complex systems. Six qualitatively different modes of information dynamics, represented in terms of their constituent atoms in the ΦID lattice.

Based on ΦID, we propose an extended taxonomy of information dynamics according to six disjoint and qualitatively distinct phenomena (Fig. 2):

**Storage**: Information that remains in the same element or set of elements (including collective effect). Comprises Red → Red, Un^1^ → Un^1^, Un^2^ → Un^2^, and Syn → Syn.

**Copy**: Information that becomes duplicated. Comprises Un^1^ → Red, and Un^2^ → Red.

**Transfer**: Information that moves between elements. Comprises Un^1^ → Un^2^ and Un^2^ → Un^1^.

**Erasure**: Duplicated information that is pruned. Comprises Red → Un^1^ and Red → Un^2^.

**Downward causation**: Collective properties that define individual futures. Comprises Syn → Un^1^, Syn → Un^2^, and Syn → Red.

**Upward causation**: Collective properties that are defined by individuals. Comprises Un^1^ → Syn, Un^2^ → Syn, and Red → Syn.

While the downward causation mode has been discussed in the past (43), upward causation and synergistic storage (Syn→Syn) have, to our knowledge, not been reported in the literature. In effect, while methods such as PID or multivariate Granger causality can effectively deal with multivariate target variables (44), they cannot untangle how each component of the target may be differently affected, and—more importantly—how sources may affect the target as a whole, without (or in addition to) affecting its parts. Overall, this taxonomy leads to less ambiguous and fully quantifiable descriptions of information dynamics in complex systems, in addition to grounding abstract concepts such as upward and downward causation,^†^ and notions such as integrated information—as we discuss below.

### A Simple Example of Information Decomposition with ΦID.

As a first example of the kind of insight that Integrated Information Decomposition can provide, let us focus on the decomposition of a variable’s so-called active information storage (AIS) (18, 48), which is defined as the TDMI of an individual part of the system (i.e. the mutual information between the present of one variable, $X_{t}^{1}$, and its own future, $X_{t+1}^{1}$). To decompose AIS, consider that in PID the mutual information of a single source variable with the target is decomposed as the sum of redundancy (which is information that each source has about the target) and that source’s own unique information. Similarly, in ΦID AIS is decomposed in terms of redundancy and unique information, but now taking into account both past and future:

[2] $$\begin{array}{cc} \text{AIS}\left(X^{1}\right)=I\left(X_{t}^{1};X_{t+1}^{1}\right) & =\text{Red}\to \text{Red}\;+\;\text{Red}\to {\text{Un}}^{1}\;\\ & +{\text{Un}}^{1}\to \text{Red}\;+\;{\text{Un}}^{1}\to {\text{Un}}^{1}\;. \end{array}$$

Here, Red→Red corresponds to redundant information in the past of both parts that is present in the future of both parts; $\text{Red}\to {\text{Un}}^{1}$ is the redundant information in the past that is eliminated from the second element and hence is only conserved in $X_{t+1}^{1}$; and similarly for the remaining atoms.

Even with this simple example, ΦID already yields insights into the system’s information dynamics: Note that although $X_{t}^{2},X_{t+1}^{2}$ are not in this mutual information, $I(X_{t}^{1};X_{t+1}^{1})$ shares the Red→Red term with $I(X_{t}^{2};X_{t+1}^{2})$ by virtue of them being considered part of the same multivariate stochastic process. Therefore, if one uses simple mutual information as a measure of storage one may include information that is not stored exclusively in a given variable, resulting in a “double-counting” of the Red→Red atom which can lead to paradoxical conclusions: The sum of individual information storages exceeding the total information flow (TDMI).

**Fig. 3.**
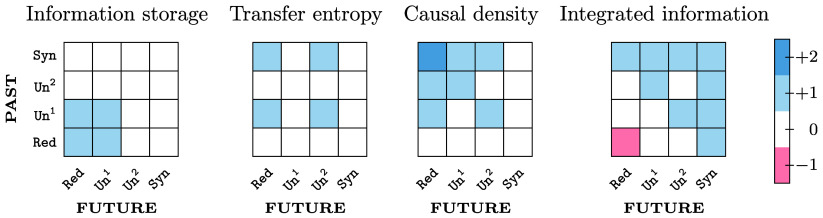
Common information-theoretic measures decomposed into integrated information atoms. Constituent ΦID atoms of active information storage, transfer entropy (from $X_{t}^{1}$ to $X_{t+1}^{2}$), causal density (sum of transfer entropies), and whole-minus-sum integrated information, highlighted in blue. Dark blue indicates double-counting, and red indicates a negative contribution (see text for details).

More generally, ΦID can be used to decompose many other quantities of interest for complex systems analysis (Fig. 3). Such decompositions can help us both to understand existing measures and design new ones. In the following sections, we apply this line of reasoning and the ΦID framework to two prominent scenarios in complex systems analysis: the assessment of causal interactions between system components and the quantification of system-wide integrated information.

## Theoretical Implications

### Different Ways of Integrating Information.

Measures of integrated information, usually denoted by Φ, aim to quantify the degree to which the temporal evolution of a dynamical system depends on the interdependencies between its parts (7),^‡^ (see ref. 12 for a review). Integrated information measures have been applied widely, most notably in the neuroscience of consciousness, but also to studies of diverse complex systems (49–51). In this section, we investigate the concept of integrated information through the lens of ΦID.

ΦID reveals that there are multiple qualitatively different ways in which a multivariate dynamical process can integrate information—through different combinations of redundant, unique, and synergistic effects. To illustrate this, we focus on the so-called “whole-minus-sum” empirical integrated information metric (52), which for a simple 2-component system is calculated as^§^

**Fig. 4.**
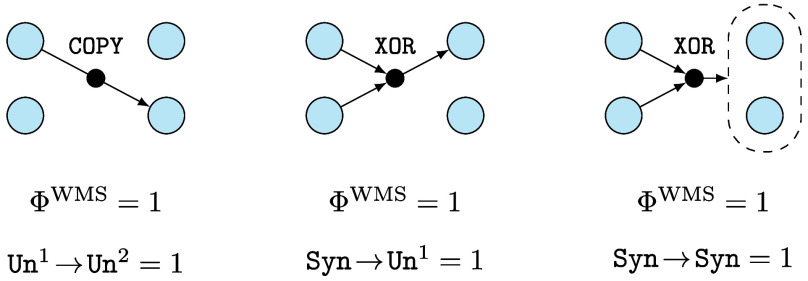
Example systems of logic gates. While these three systems have the same integrated information (measured with $\Phi^{\text{WMS}}$), their information dynamics are radically different. The idiosyncrasy of each type of dynamic is captured by the ΦID formalism, which shows that in each system there is only one nonzero atom, different for each system.

[3] $$\begin{array}{c} \Phi^{\text{WMS}}=I\left(X_{t};X_{t+1}\right)-\sum_{i}I\left(X_{t}^{i};X_{t+1}^{i}\right)\;. \end{array}$$

which reflects a balance between the information contained within the whole system ($I(X_{t};X_{t+1})$) and the information contained within the parts ($I(X_{t}^{1};X_{t+1}^{1})$ and $I(X_{t}^{2};X_{t+1}^{2})$). We apply this metric to the following elementary examples of 2 binary variables (Fig. 4):

- A copy transfer system, in which $X_{t}^{1},X_{t}^{2},X_{t+1}^{1}$ are i.i.d. fair coin flips, and $X_{t+1}^{2}=X_{t}^{1}$ (i.e. the information of $X_{t}^{1}$ is copied to $X_{t+1}^{2}$).
- The downward XOR, in which $X_{t}^{1},X_{t}^{2},X_{t+1}^{2}$ are i.i.d. fair coin flips, and $X_{t+1}^{1}\equiv X_{t}^{1}+X_{t}^{2}\;\text{(mod}\text{2)}$.
- The parity-preserving random (PPR), in which $X_{t}^{1},X_{t}^{2}$ are i.i.d. fair coin flips, and $X_{t+1}^{1}+X_{t+1}^{2}\equiv X_{t}^{1}+X_{t}^{2}\;\text{(mod}\text{2)}$ (i.e. $X_{t+1}$ is a random string of the same parity as $X_{t}$).

Direct calculation shows that these three systems are “equally integrated:” $\Phi^{\text{WMS}}=1$ for all of them, which implies that the degree to which the dynamics of the whole cannot be perfectly predicted from the parts alone is equivalent (12, 52). However, a more nuanced analysis using ΦID reveals that these systems integrate information in qualitatively different ways. In effect, the integration in the copy system is entirely due to transfer dynamics (${\text{Un}}^{1}\to {\text{Un}}^{2}$); the downward XOR integrates information by transforming synergistic into unique information ($\text{Syn}\to {\text{Un}}^{1}$); and PPR due to persistent synergy in the past and future (Syn→Syn). All the other ΦID atoms in each system are zero (proofs in *SI Appendix*).

### Measures of Integrated Information Capture Multiple ΦID Atoms.

Within the IIT literature, researchers have proposed multiple measures aimed at quantifying to what extent a system is integrated as a whole, in terms of its parts influencing each other’s evolution over time (53). These measures, though superficially similar, are known to behave inconsistently, for reasons that are not always clear (12). Here, we use ΦID to dissect and compare three existing measures of integrated information ($\Phi^{\text{WMS}}$, ψ, $\Phi_{G}$) and CD, bringing to light their similarities and differences.^¶^

To perform a systematic characterization, one can determine which measures are sensitive to which modes of information dynamics by calculating whether each measure is zero, positive, or negative for a system consisting of only one particular ΦID atom (Table 1; proofs in *SI Appendix*). By applying this strategy across different measures of integrated information, we find that each proposed measure captures a distinct combination of ΦID atoms: Although generally most of them capture synergistic effects and avoid (or penalize) redundant effects, they differ substantially.

**Table 1.** Sensitivity of integrated information measures to each ΦID atom

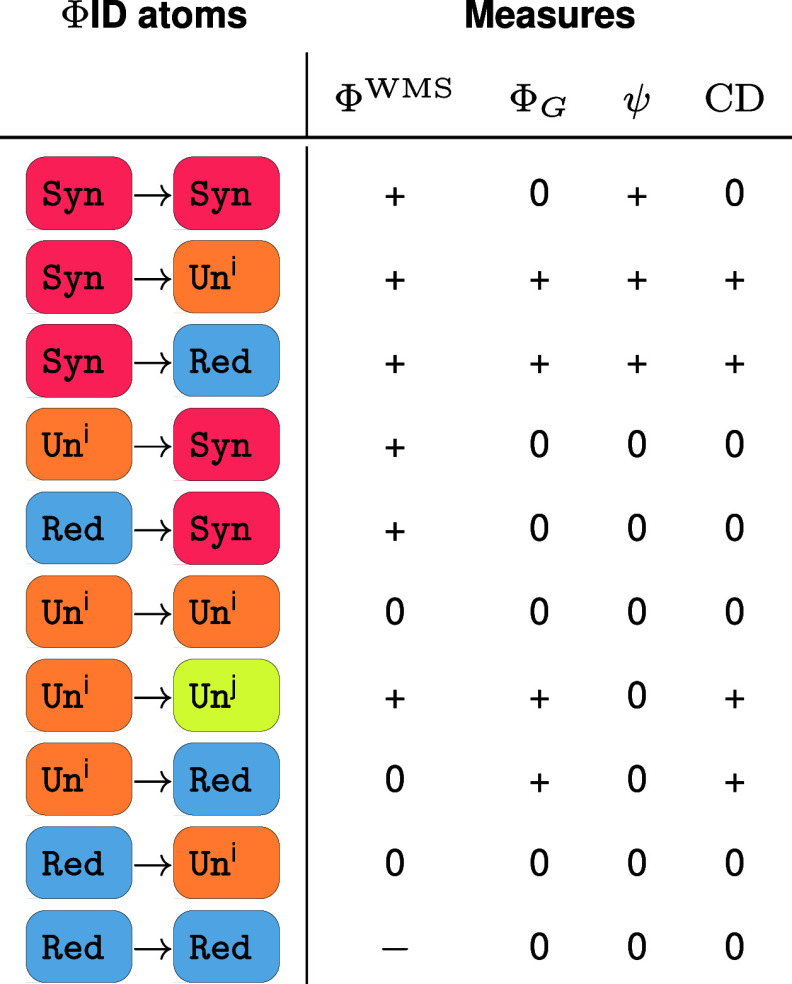

For each measure, entries indicate whether the value is positive (+), negative (−), or 0 in a system in which the given ΦID atom is the only nonzero atom. Atom color code taken from Fig. 1.

The conclusion of this analysis is that these measures are not simply different approximations of a single concept of integration, but rather they are capturing intrinsically different aspects of the system’s information dynamics. While aggregate measures like these can be empirically useful, one should keep in mind that they are measuring combinations of different effects within the system’s information dynamics. Echoing the conclusions of ref. 12: These measures behave differently not only in practice but also in principle.

### A ΦID Account of Information Transfer.

Most methods of statistical causal discovery use the conditional mutual information^#^ as their main building block. Here, we illustrate how the widely used transfer entropy (TE) (15) can be decomposed in terms of ΦID, showing that it conflates qualitatively distinct effects in nonstraightforward ways. The TE from the system’s first to its second element, defined as $\text{TE}\left(1\to 2\right):=I\left(X_{t}^{1};X_{t+1}^{2}|X_{t}^{2}\right)$, can be decomposed as

[4] $$\begin{array}{c} \begin{array}{cc} \text{TE}(1\to 2) & =\text{Syn}\to \text{Red}\;+\;\text{Syn}\to {\text{Un}}^{2}\\ & +{\text{Un}}^{1}\to \text{Red}\;+\;{\text{Un}}^{1}\to {\text{Un}}^{2}\;. \end{array} \end{array}$$

Of these, ${\text{Un}}^{1}\to {\text{Un}}^{2}$ is the only “genuine” transfer term: All others correspond to redundant or synergistic effects involving both variables in past or future, which do not reflect any kind of transfer phenomena. This complicates the interpretation of TE as a fully general measure of information transfer.^‖^ Crucially, PID decomposition of the TE (55) cannot separate genuine transfer from duplication. Thanks to ΦID, we can isolate the part of TE that corresponds to genuine information transfer (${\text{Un}}^{1}\to {\text{Un}}^{2}$).

Additionally, Eq. **4** implies that the atom Syn→Red is accounted for in both TE(1→2) and TE(2→1). This has an important consequence: If one seeks to quantify the total causal influence within the system via adding up both TEs [a quantity known as unnormalized causal density (uCD) (12)], this may overestimate the interdependencies by double-counting this atom (Fig. 3). This overestimation is a known shortcoming of uCD, potentially exceeding the total TDMI (16). ΦID reveals the reason for this overestimation (double-counting of Syn→Red), and provides a practical solution: We can correct uCD by subtracting the double-counted Syn→Red atom. Solving this problem may have important consequences in fields such as neuroscience, where the total TE of a brain region is a popular metric of hierarchical organization (56, 57).

The decomposition of uCD via ΦID reveals another limitation of traditional causal discovery methods: They do not explicitly account for modes of information flow that involve synergy in the targets. Therefore, while these methods account for possible interactions of source variables, they neglect interactions in the targets—which are not considered jointly. As an example, take the parity-preserving system in Fig. 4: This system has zero TE and zero AIS, yet it clearly has dynamical structure (as picked up by $\Phi^{\text{WMS}}$). This is an example of information being carried purely in high-order effects, in a way that common measures like AIS and TE are unable to capture. More generally, we expect this to be particularly prevalent in systems with distinct micro- and macroscale behavior (45, 46, 58).

### Popular Measures of Information Storage and Transfer Reflect Overlapping Phenomena.

We have already seen that transfer entropy is not a monolithic quantity, instead comprising four distinct types of information dynamics (Fig. 3). Crucially, observing nonzero TE is not diagnostic of which of its constituent atoms is present. This is because these four distinct atoms do not all need to be present together: The presence of any one of them will give rise to nonzero TE—and be interpreted as information transfer, despite the underlying phenomena being actually very heterogeneous. The same is also true for AIS: Nonzero AIS can also arise from any combination of four distinct ΦID atoms, and simply observing nonzero AIS will not provide insight about which phenomenon is responsible for the observed storage.

Crucially, information duplication (${\text{Un}}^{1}\to \text{Red}$) is present in the conceptual decompositions of both TE and AIS. For AIS, ${\text{Un}}^{1}\to \text{Red}$ fits the definition of “information that was present in $X^{1}$ at time t, and is present in $X^{1}$ also at t+1” (because at t+1 it is present in both $X^{1}$ and $X^{2}$). For TE, ${\text{Un}}^{1}\to \text{Red}$ fits the definition of ‘information that was present in $X^{1}$ at t, and is present in $X^{2}$ at t+1” (because at t+1 it is present in both $X^{1}$ and $X^{2}$).

Since ${\text{Un}}^{1}\to \text{Red}$ is sufficient for both AIS or TE, but necessary for neither, in a system without any ${\text{Un}}^{1}\to \text{Red}$ it is still possible to observe both nonzero AIS and nonzero TE, and for them to be even correlated. However, an observed correlation could also be driven by presence of nonzero ${\text{Un}}^{1}\to \text{Red}$. In the extreme case of a system where the only type of information dynamics is duplication (${\text{Un}}^{1}\to \text{Red}$), TE and AIS will be perfectly correlated: literally two names for the same phenomenon.

This realization has direct implications for both empirical and computational studies, where TE and AIS are routinely treated as independent read-outs of the system’s behavior and compared against each other (59–61). To what extent do such observations reflect genuinely different phenomena, versus two names for one and the same phenomenon? ΦID allows to disambiguate these possibilities. By isolating the ${\text{Un}}^{1}\to \text{Red}$ atom, thanks to ΦID we can precisely quantify to what extent it drives the correlation between transfer entropy and active information storage.

## Applications to Empirical and Simulated Data

In this section, we showcase three applications of ΦID to simulated and real data, illustrating the capabilities of ΦID to yield new insights and solve practical problems. In each application, we calculate ΦID using the joint distribution $p(X_{t},X_{t+1})$ estimated from the empirical data. As stated above, ΦID inherits PID’s limitation of requiring the additional specification of a double-redundancy function to enable the calculation of ΦID atoms. As in the case of PID, this underdetermination gives room to a range of options (see e.g. refs. 17, 37, 62, and 63). In all examples below, we use a multitarget extension of Barrett’s MMI measure, into which many other PIDs collapse for the case of Gaussian variables (63). A discussion of MMI and its defining features, limitations, and applicability is presented in *SI Appendix*. For completeness, we also show that the empirical results replicate with a multitarget extension of Ince’s Common Change in Surprisal (CCS) measure (62) (*SI Appendix*, Figs. S1–S5).

### Why Whole-Minus-Sum Φ Can Be Negative.

ΦID can also explain certain perplexing behaviors of integrated information and dynamical complexity measures. In particular, $\Phi^{\text{WMS}}$ can sometimes take negative values, giving rise to the counterintuitive notion of a “negatively integrated” system. This behavior has been used as an argument to discard $\Phi^{\text{WMS}}$ as a suitable measure of integrated information (16, 54). ΦID sheds light on this paradoxical behavior, and furnishes a simple solution.

By applying ΦID to the definition of $\Phi^{\text{WMS}}$ in Eq. **3**, one finds that $\Phi^{\text{WMS}}$ accounts for all the synergies in the system, the unique information transferred between parts of the system and, importantly, the negative of the Red→Red atom (Fig. 3; see *SI Appendix* for details). The presence of this negative double-redundancy term shows that $\Phi^{\text{WMS}}$ can be negative in highly redundant systems, in which Red→Red is larger than all other atoms that constitute $\Phi^{\text{WMS}}$. This is akin to Williams and Beer’s (17) explanation of the negativity of the interaction information (64), applied to multivariate processes. Based on this insight, one can formulate a “revised” measure of integrated information, $\Phi^{\text{R}}$, by adding back the double-redundancy, which includes only synergistic and unique transfer terms. In terms of mutual information, MMI-$\Phi^{\text{R}}$ is obtained for a bipartite system as

$$\begin{array}{c} \Phi^{\text{R}}=I\left(X_{t};X_{t+1}\right)-\sum_{i=1}^{2}I\left(X_{t}^{i};X_{t+1}^{i}\right)+\min_{i,j}I\left(X_{t}^{i};X_{t+1}^{j}\right). \end{array}$$

**Fig. 5.**
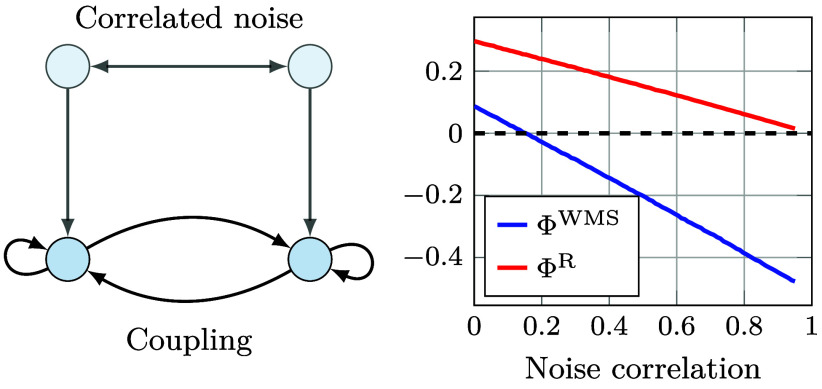
Standard and revised $\Phi^{\text{WMS}}$ in a two-component noisy autoregressive system. As the noise injected to both components becomes more correlated, $\Phi^{\text{WMS}}$ drops below zero while $\Phi^{\text{R}}$ remains positive.

We computed $\Phi^{\text{R}}$ numerically for a simple two-node autoregressive (AR) system, mimicking the setting in ref. 12. The system consists of two continuous variables with dynamics such that $X_{t+1}∼N\left(AX_{t};\Sigma \right)$, with A being a 2×2 matrix with all entries set to 0.4, and Σ a noise (or innovations) covariance matrix with 1s along the diagonal and a given noise correlation (c in the notation of ref. 12) in the off-diagonal entries. We calculated $\Phi^{\text{WMS}}$ and $\Phi^{\text{R}}$ with respect to the system’s stationary distribution, which can be shown to be a multivariate Gaussian. Plots of both quantities are shown in Fig. 5, and details of the computation can be found in *SI Appendix*.

As expected, $\Phi^{\text{WMS}}$ drops below zero as synergy decreases and redundancy increases with noise correlation. However, after adding back the double-redundancy term, the revised version, $\Phi^{\text{R}}$, tends to 0 for high noise correlation, which is more consistent with some of the other measures highlighted in ref. 12, e.g. CD and $\Phi^{*}$.

### Information Decomposition in Simulated Whole-Brain Activity.

To demonstrate that these empirical results also apply to more complex scenarios beyond toy models, we next analyze simulated whole-brain activity via the Dynamic Mean Field (DMF) model (65). This model simulates human fMRI signals by representing cortical regions as macroscopic neural fields, whose local dynamics are described by a set of coupled differential equations. The DMF model incorporates realistic aspects of neurophysiology such as synaptic dynamics and membrane potential (66–68), and is informed by the empirical network of anatomical connections obtained from diffusion tensor imaging (DTI) - while having the advantage of being free from physiological noise confounds. We simulate the DMF equations using a DTI-based connectome obtained from the Human Connectome Project using the same model settings as (69), and compute $\Phi^{\text{WMS}}$ and $\Phi^{\text{R}}$ for all pairs of brain regions.^**^ These values were calculated for varying values of a global coupling parameter (denoted by G) and the resulting average values (over all pairs of brain regions) were then analyzed. The details of the model, the simulation procedure, and the computation of integrated information measures can be found in *SI Appendix*.

**Fig. 6.**
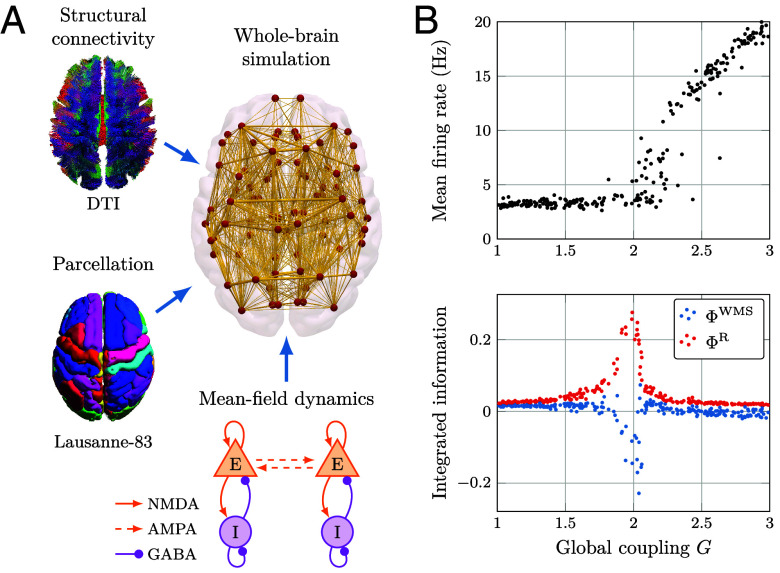
Measures of integrated information in a whole-brain computational model. (*A*) Schematic diagram of the Dynamic Mean-Field (DMF) model (65, 71), which combines a DTI-based connectome and a whole-brain parcellation to simulate realistic firing rates; a hemodynamic model (72) transforms the firing rates into BOLD signals. (*B*) As the global coupling parameter G is increased, the mean firing rate exhibits a sharp increase at approximately G=2. Importantly, $\Phi^{\text{WMS}}$ takes negative values, suggesting a conceptually problematic negative value of integration—while the revised measure $\Phi^{\text{R}}$ shows a strong positive peak and is nonnegative.

As shown in Fig. 6, for values of G close to 2, the mean firing rate of the model increases sharply. Around this point $\Phi^{\text{WMS}}$ becomes sharply negative, which would correspond to the conceptually problematic notion of a system that is “negatively integrated.” Crucially, when the double-counting of redundancy is corrected and $\Phi^{\text{R}}$ is used instead of $\Phi^{\text{WMS}}$, a different picture appears: Integration (understood as synergy plus transfer) is always positive, and it strongly increases and peaks in the region around G=2. This result aligns well with prior literature (69, 70) showing that the point around G=2 corresponds to the model’s optimal fit to data from awake subjects, and that a high level of integration is required for the normal operation of the brain in healthy, conscious individuals (6).

The strong discrepancy between $\Phi^{\text{WMS}}$ and $\Phi^{\text{R}}$ confirms that the concerns regarding the conflation of multiple information effects highlighted throughout this article are not a mere theoretical issue but can trigger misleading interpretations in the analysis of neuroscientific data. Together, Figs. 5 and 6 show how ΦID can disambiguate between qualitatively different dynamical phenomena in time series data, and resolve theoretical difficulties of well-known measures, not only in theory but also in practical applications.

### ΦID Sheds Light on Empirical Results.

To provide an empirical demonstration of the capabilities of ΦID, we use it to study the dynamical relationship that exists between heart rate and respiration in healthy humans. This choice is motivated by the well-known influence of respiration on heart rate, which can be captured in terms of transfer entropy between respiratory volume and heart rate time series (73–75). Therefore, we seek to decompose this effect into its constituent information-dynamic elements.

**Fig. 7.**
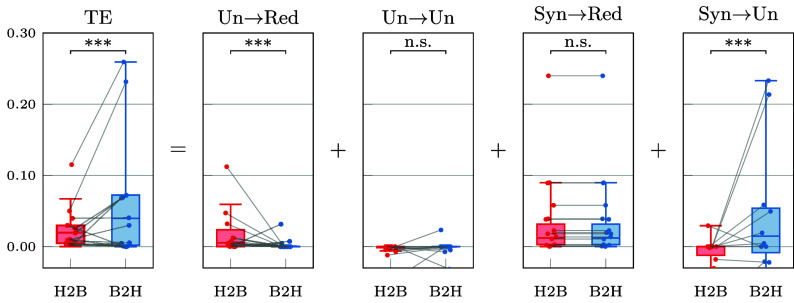
Decomposition of information transfer between heart rate and respiratory volume. Transfer entropy is decomposed into its four constituent ΦID atoms, calculated in both directions (heart to breath [H2B] and breath to heart [B2H]).

Specifically, we analyze the *Fantasia* database (76), an openly available dataset that contains data from 40 healthy subjects while watching the Disney movie “Fantasia.” Following the preprocessing pipeline outlined in ref. 73, we extracted synchronized time series for interbeat intervals from the ECG timeseries, and detrended the respiratory volume. Using the resulting data, we calculate both the TE from heart to breath and from breath to heart, and then proceed to decompose these quantities in terms of their ΦID constituents (Fig. 7).

As expected based on previous work, the TE from breath to heart is significantly higher than from heart to breath. Crucially, our analysis reveals that this effect is driven by two distinct ΦID atoms, out of the four that comprise transfer entropy. The TE result is dominated by the Syn→Un atom, while the transfer atom itself (Un→Un) shows no significant differences. Importantly, however, Un→Red shows a significant effect in the opposite direction to the main TE result. The standard TE analysis is unable to resolve these modes of information dynamics, and thus misses the heart’s unique contribution to the heart-breath joint dynamics.

In summary, ΦID shows that the effect seen in the TE toward the heart is not pure transfer but synergistic downward causation, and that there is a smaller information duplication effect originating at the heart that is overshadowed by the former. These empirical results illustrate the type of insight that ΦID can bring beyond standard transfer entropy and Granger causality analyses.

### Explaining Empirical Results on Information Storage and Transfer.

**Fig. 8.**
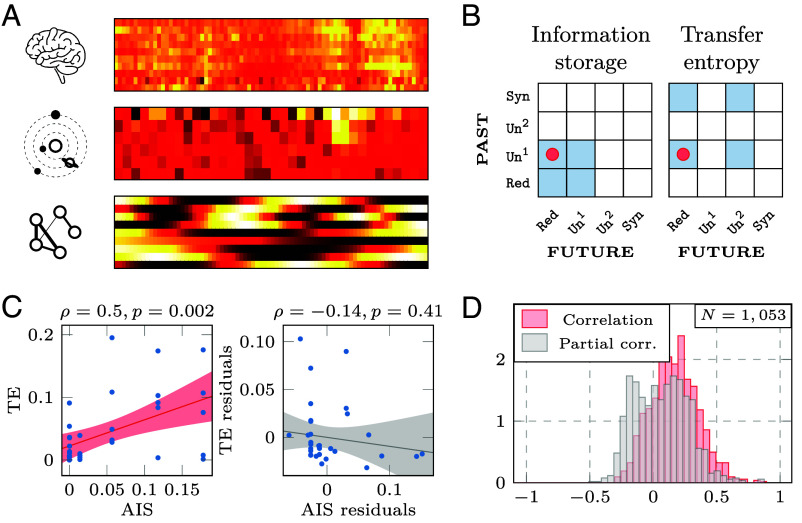
Integrated information decomposition of 1,053 dynamical systems. (*A*) Example of time-series data from different dynamical systems: neuroscience (functional MRI data of human brain regions); astrophysics (light curves of astronomical objects from the Photometric LSST Astronomical Time Series Classification Challenge); and from a synthetic model (coupled oscillators). (*B*) Integrated information decomposition of active information storage and transfer entropy shows that both measures include duplication of information (${\text{Un}}^{1}\to \text{Red}$). (*C*) Example plots of the statistical association between AIS and TE, before (*Left*) and after (*Right*) regressing out the ${\text{Un}}^{1}\to \text{Red}$ atom. It is clear that for this system (system 388 from the pyspy database), the significant association between AIS and TE is driven by the shared presence of information duplication in both measures. (*D*) Histogram of statistical association between AIS and TE across all 1,053 dynamical systems in the pyspy database; red indicates simple correlation, and gray indicates partial correlation correcting for information duplication (${\text{Un}}^{1}\to \text{Red}$). A notable shift in distribution is visible, such that regressing out the influence of the shared atom significantly reduces the mean association between TE and AIS (Cohen’s d=0.76; $P<10^{-5}$; 83% of systems have lower partial correlation than correlation).

To complement our theoretical insight about the conceptual relationship between TE and AIS, and demonstrate its empirical relevance, we analyze N=1,053 datasets of multivariate time-series from a wide variety of biological, physical, social, and synthetic systems Cliff et al. (36) (Fig. 8*A*). A full description is available in ref. 36. Each dataset comprises up to 2,000 observations from 5 to 40 variables.

For each dataset, we compute the Gaussian ΦID decomposition between each pair of variables (X and Y), and then obtain TE from X to Y, and AIS within X, as per our decomposition (Fig. 8*B*). Thus, we obtain one value of TE and one value of AIS for each variable in each of our 1,053 datasets. Within each dataset, we correlate AIS and TE across variables, thereby obtaining a distribution of 1,053 AIS-TE correlations (Fig. 8*D*). We also obtain the partial correlation between AIS and TE within each of the 1,053 datasets, after partialling out the influence of the ${\text{Un}}^{1}\to \text{Red}$ information atom, which is shared by AIS and TE. That is, within each system we quantify to what extent the relationship between AIS and TE is driven by the presence of an information atom in common. We find that across the whole set of 1,053 empirical and synthetic multivariate systems, there is on average a positive correlation between TE and AIS (mean = 0.17; Fig. 8*D*). However, this relationship is significantly diminished to near-zero (mean =−0.001) once the effect of the common information atom ${\text{Un}}^{1}\to \text{Red}$ is removed (t=24.5, $P<10^{-5}$).

Thus, on average there appears to be a positive relationship between traditional measures of information storage and transfer, across a wide variety of real-world and synthetic systems. However, this correlation is largely driven by the fact that both measures include information duplication (${\text{Un}}^{1}\to \text{Red}$) as one of their components—meaning that it could be misleading to treat AIS and TE as independent, without taking into account the shared role of information duplication. Fig. 8*C* shows an example of a system where TE and AIS appear to be strongly positively correlated (ρ=0.50, P=0.02), but the correlation is entirely due to the shared role of information duplication: The two measures become clearly independent (ρ=−0.02, P=0.87) once the role of information duplication is partialled out.

To be clear, this is not to say that AIS and TE cannot be related: On the contrary, even after information duplication is partialled out we observe a broad distribution of both positive and negative correlations between AIS and TE, across the 1,053 systems under consideration. This is reasonable, since each of TE and AIS includes several information-dynamic phenomena beyond information duplication. However, without recognizing the presence of a shared atom (information duplication) in AIS and TE thanks to ΦID, an analysis like the one in Fig. 8*D* could have led to the conclusion that on average, systems that transmit more information also tend to store more information, as indicated by a positive average correlation between AIS and TE across systems. In fact, we see that such a conclusion would be misleading, due to the fact that both AIS and TE reflect information duplication. This finding is only made possible by ΦID, since the theoretical relationship between TE and AIS is invisible to traditional PID: One needs to be able to take into account multiple sources and multiple targets at the same time, as ΦID does, in order to discern the overlapping atom of information duplication.^††^

Our empirical analysis reveals that TE-AIS correlations are ubiquitously driven by information duplication.This insight about empirical behavior of information-dynamic measures is consistent when instead of the MMI definition of double-redundancy, we use the CCS operationalization of double-redundancy (*SI Appendix*, Fig. S4). In *SI Appendix*, we also show that across the 1,053 dynamical systems of the pyspy database, there is a strong and significant positive correlation between $\Phi^{\text{R}}$ quantified using CCS and using MMI (Spearman’s ρ = 0.95, P<0.001; *SI Appendix*, Fig. S5), reflecting the robustness of our approach.

## Discussion

This paper introduces ΦID as a formal framework to study high-order interactions in the dynamics of multivariate complex systems, and decompose multivariate information flow into interpretable, distinct parts. This decomposition brings two important outcomes. First, it allows us to better understand and refine existing metrics of information exchange and dynamical complexity. Second, it enables systematic analyses of previously unexplored modes of information dynamics.

### Toward Multidimensional Accounts of Dynamical Complexity.

ΦID provides principled tools to inspect existing measures of information dynamics and overcome some of their shortcomings. In particular, we have shown that both the widely used transfer entropy, active information storage, and what is referred to as “integrated information” in the context of IIT are in fact aggregates of several distinct information effects, typically including transfer and synergy phenomena. In addition, our analysis shows that different measures of integrated information actually capture different ΦID atoms in various proportions, which provides a formal explanation for the heterogeneity among existing measures from ref. 12.

Supporting our theoretical results with practical relevance, we showed in empirical physiological data that significant differences in transfer entropy can be observed even in the absence of genuine information transfer phenomena. Likewise, our analysis based on whole-brain modeling showed how the conflation of distinct information phenomena in existing measures of integrated information can introduce substantial confusion in the results and interpretation of neuroscientific analysis. ΦID provides tools to identify the conceptual roots of these problems, and also to fix them, by tailoring measures that target the specific kinds of information dynamics one wishes to analyze.

As well as providing a taxonomy of information dynamics phenomena, ΦID establishes that there are fundamental limitations to any purported all-encompassing scalar measure of dynamical complexity, in line with Feldman and Crutchfield (77). The space of possible complex dynamics is, unsurprisingly, vast and complex, and while scalar measures might still have great practical value in specific contexts,^‡‡^ΦID clarifies that a general theory of complex systems (biological or otherwise) cannot be reduced to a single, one-size-fits-all measure, but rather needs to embrace this richness.

### Disentangling Common Dynamics of Information Storage and Transfer.

PID provided insight about TE by revealing that it can comprise synergistic information (17). Being able to take into account both multiple sources and multiple targets simultaneously, ΦID adds to this insight by showing that even the nonsynergistic part of TE is not a single type of information-dynamic, instead comprising pure transfer from x to y, but also ${\text{Un}}^{1}\to \text{Red}$: duplication of the information held in x. Notably, ${\text{Un}}^{1}\to \text{Red}$ can also be considered as a type of information storage, being part of AIS.

This means that, while it is true that the formulation of TE “specifically eliminates information storage in the past of the target process from being mistakenly considered as having been transferred” (79), nevertheless TE still includes some form of storage. Since ${\text{Un}}^{1}\to \text{Red}$ is part of TE, we can also conclude the converse: that AIS involves some form of information transfer.

### Limitations and Future Work.

Naturally, ΦID inherits many characteristics and limitations of PID. In particular, ΦID specifies the relationships between information atoms, but does not prescribe a particular functional form for them. To compute numerical values of ΦID atoms one needs a multitarget extension of PID’s redundancy function. The PID literature has proposed several valid but distinct redundancy functions, arising from different fields such as neuroscience, cryptography, or game theory, each with their own specific desiderata. While many of these formulations have been shown to agree in various empirical scenarios (38), some of these desiderata are mutually incompatible (80), suggesting that none of them is universally preferable (81). This implies that practitioners need to choose which PID to use based on domain knowledge and characteristics of the data and problem at hand (e.g., some PIDs are only defined for discrete data)—with the awareness that empirical results may not always generalize across different PIDs.

It is useful to contemplate the similarities between this feature of PID (which ΦID inherits) and Bayesian statistics. The empirical application of Bayesian statistics requires the specification of a prior, but there is no single prior that is universally agreed-upon for all situations: Practitioners need to choose an appropriate prior based on domain-expertise and the research question at hand, with the awareness that empirical results may not always hold when the prior changes. Further analyses can assess the degree to which results are robust to the choice of prior (82). In this spirit, we advocate for demonstrating that empirical findings using PID and ΦID hold across different redundancy formulations. For example, Barrett (63) showed that many PID formulations converge to MMI (the main one used here) in the case of Gaussian systems. Recent work also showed that the CCS measure that we use is in good agreement with at least 2 other PID formulations ($I_{\text{BROJA}}$ and $I_{\text{dep}}$) in both real and simulated neuronal data (83). Following this principle, here we showed that our empirical results could be replicated across MMI and CCS redundancies (*SI Appendix*, Figs. S1–S5), with $\Phi^{\text{R}}$ exhibiting a correlation of 0.95 across 1,053 dynamical systems when computed with MMI or CCS. While this is no guarantee that the same results will generalize across all possible double-redundancy functions, it does demonstrate that they are not specific to one particular ΦID formulation. Going forward, the formulation of novel double-redundancy functions that satisfy different desiderata, and a thorough comparison in simulated systems (following refs. 12 and 83) are important lines for future work. Likewise, future work could explore whether the ΦID framework yields dynamical insights to further clarify the desirable properties of redundancy functions, potentially helping to address some current challenges of PID.

More broadly, it is important to distinguish between the formal (mathematical) decomposition of information-theoretic quantities afforded by PID and ΦID, and the numerical values assigned to each atom based on specific data and a specific choice of redundancy function. Different PID formulations may disagree on the specific numerical values assigned to each atom, but they all share the same formal decomposition. Accordingly, our results pertaining to the formal decomposition of $\Phi^{\text{WMS}}$, and the demonstration that the decompositions of AIS and TE overlap (Fig. 3), only rely on the theoretical aspects of ΦID, not on the specific redundancy formulation.

ΦID is a conceptual scaffold to decompose multivariate mutual information, and the nature of the resulting decomposition critically depends on how the underlying joint distribution has been constructed. In particular, note that the ΦID framework depends only on a joint probability distribution $p(X_{t},X_{t+1})$, and thus its results can be interpreted as causality in the Pearl or Granger sense, depending on whether the distribution comes from intervention or observation, respectively. If the distribution is built on observational data then the decomposition generally should be understood in the Granger-causal sense (i.e. as referring to predictive ability). Similarly, if the conditional distribution $p(X_{t+1}|X_{t})$ is equivalent to a do() distribution in Pearl’s sense (84), and the system satisfies the faithfulness and causal Markov conditions, then the results of ΦID are to be interpreted in a counterfactual causal sense. In either case, the formalism developed here applies directly.

Additionally, given that the analyses presented here are based on an empirical joint distribution $p(X_{t},X_{t+1})$ directly estimated from data, the corresponding results refer to what the system does (the system’s behavior)—in contrast to how the system is structured (i.e., the underlying mechanisms that causally explain its behavior, as study e.g. in ref. 85). Note that this limitation is not specific to ΦID or PID: It is inherent to any analysis based on observational data. Crucially, although it may be expected that high-order behavior requires the presence of underlying high-order mechanisms (e.g., dynamical laws involving beyond-pairwise interactions), this intuition is misleading. In fact, it has been shown that even in the absence of high-order mechanisms, systems can still exhibit complex high-order behaviors (86), and hence a complete account of higher-order phenomena needs to embrace both. By disentangling different types of information dynamics and enabling their quantification in empirical data, ΦID enhances our ability to scrutinize high-order behaviors. That being said, ΦID also can be used to investigate mechanisms, for example by constructing the joint distribution $p(X_{t},X_{t+1})$ using a description of an input–output system in the form of a conditional probability $p(X_{t+1}|X_{t})$ and a distribution of inputs $p(X_{t})$. This usage of ΦID complements other recent approaches to study high-order mechanisms in complex systems (85, 87).

Finally, it is important to note that the framework presented in this paper focuses on decomposing the mutual information in processes considered at two time points. While this captures all the information carried in Markovian systems, it might miss important phenomena in systems with non-Markovian dynamics. Given the importance of non-Markov scenarios, which naturally arise in settings with nonobservable variables, an important direction for future work is to investigate the effect of unobserved variables on the proposed decomposition. This could be done, e.g., by leveraging Taken’s embedding theorem or other methods (88, 89).

Overall, ΦID generalizes PID to many-to-many interactions. It provides insights about widely used measures of information storage, transfer, integration, and complexity, which are consistent across different operationalizations (MMI, CCS) and across a broad range of empirical data. While ΦID inherits some inherent limitations of PID, and we look forward to future refinement, we believe that it can provide the basis for fruitful avenues of theoretical and empirical discovery.

## Materials and Methods

This section establishes the mathematical bases of our framework. The aim is to build a decomposition of the TDMI, as defined in Eq. **1**, that differentiates the role of each source and each target (possibly multivariate) variable simultaneously, as illustrated in Fig. 1*A*. By doing this, we overcome PID’s limitation of having only one single target variable and formulate a multitarget information decomposition. In the context where the sources and targets are past and future of two time-series, this formulation corresponds to identifying how the two variables change their way of carrying information over time. Thus, ΦID atoms can be represented by a matrix, where each atom corresponds to a combination of redundant, unique, or synergistic information in the past and future. In *SI Appendix*, we provide an alternative explanation of our formalism in terms of the decompositions enabled by a forward PID (where variables at time t and t+1 are sources and targets, respectively) and a backward PID (where the assignment of sources and targets is reversed). Note that throughout this section, we switch from the Red/Un/Syn notation above to the more standard (and more general) “curly bracket” notation introduced by Williams and Beer (17).

### Double-Redundancy Lattice.

Let us start by reviewing the construction of the redundancy lattice that is employed in PID to formalize our intuitive understanding of redundancy (17). This lattice is built over the set A, which for the case of two time series can be expressed as

[5] $$\begin{array}{c} A:=\{\{1\},\{2\},\{1,2\},\{\{1\},\{2\}\}\}, \end{array}$$

which correspond to all the sets of subsets of {1,2}, where no element is contained in another.^§§^ Then, the lattice is built using a natural (partial) order relationship that exists between the elements of A (17): For α,β∈A, one says that

[6] $$\begin{array}{c} \alpha ⪯\beta \;\text{if}\;\text{for all}\;b\in \beta \;\text{there exists}\;a\in \alpha \;\text{such that}\;a\subset b. \end{array}$$

The lattice that encodes the relationship ⪯ is known as the redundancy lattice (Fig. 1*B*), and guides the construction of the four terms in the PID.

Our first step in building the foundations of ΦID is to build a *product lattice* over A×A, in order to extend the notion of redundancy from PID to the case of multiple source and target variables (here $X_{t}^{1}$, $X_{t}^{2}$ and $X_{t+1}^{1}$, $X_{t+1}^{2}$ respectively). Intuitively, ΦID is the “product” of two complementary single-target PIDs, one decomposing the information carried by the past about the future, and the other decomposing the information carried by the future about the past (Fig. 1*A*). To formalize this intuition, we extend Williams and Beer’s (17) notation, and denote sets of sources and targets using their indices only with an arrow going from past to future. Hence, the nodes of the product lattice are denoted as α→β for α,β∈A.

A natural partial ordering relationship can be established over the product lattice as follows:

[7] $$\begin{array}{c} \alpha \to \beta ⪯\alpha^{\prime }\to \beta^{\prime }\;\text{iff}\;\alpha ⪯\alpha^{\prime }\;\;\text{and}\;\;\beta ⪯\beta^{\prime }. \end{array}$$

This relationship establishes a lattice structure,^¶¶^ which for the case of a bipartite system consists of 16 nodes (Fig. 1*C*).

### Redundancies and Atoms.

The other ingredient in the PID recipe—besides the redundancy lattice—is a *redundancy function*, $I_{\cap }$, that quantifies the amount of “overlapping” information about the target that is common to a set of sources α∈A (17). The redundancy function in a PID, $I_{\cap }^{\alpha }$, encompasses the following terms in the case of two source variables:

- $I_{\cap }^{\left\{1\}\{2\right\}}$ is the information about the target that is in either source,
- $I_{\cap }^{\left\{i\right\}}$ is the information in source i, and
- $I_{\cap }^{\left\{12\right\}}$ is the information that is in both sources when considered together.

This subsection extends the notion of overlapping information to the multitarget setting.

For a given α→β∈A×A, the overlapping information that is common to sources α and can be seen in targets β is denoted as $I_{\cap }^{\alpha \to \beta }$ and referred to as the *double-redundancy function*. Here, we assume that the double-redundancy function satisfies two axioms:

- Axiom 1 (compatibility): If $\alpha =\{\alpha_{1},\cdots ,\alpha_{J}\}$ and $\beta =\{\beta_{1},\cdots ,\beta_{K}\}$ with α,β∈A and $\alpha_{j},\beta_{k}$ nonempty subsets of {1,⋯,N}, then the following cases can be reduced to the redundancy of PID or the mutual information:^##^
  $$\begin{array}{c} I_{\cap }^{\alpha \to \beta }=\left\{\begin{array}{cc} \mathrm{Red}(X^{\alpha_{1}},\cdots ,X^{\alpha_{J}};Y^{\beta_{1}}) & \text{if}\;K=1,\\ \mathrm{Red}(Y^{\beta_{1}},\cdots ,Y^{\beta_{K}};X^{\alpha_{1}}) & \text{if}\;J=1,\\ I(X^{\alpha_{1}};Y^{\beta_{1}}) & \text{if}\;J=K=1. \end{array}\right. \end{array}$$
- Axiom 2 (subset equality): If $\alpha =\{\alpha_{1},\cdots ,\alpha_{J}\}$, $\beta =\{\beta ,\cdots ,\beta_{K}\}$, and $\alpha^{\prime }=\left\{\alpha_{1},\cdots ,\alpha_{J},\alpha_{J+1}\right\}$, then
  [8] $$\begin{array}{c} I_{\cap }^{\alpha^{\prime }\to \beta }=I_{\cap }^{\alpha \to \beta }\;\text{if}\;\alpha_{J}\subseteq \alpha_{J+1}. \end{array}$$
  Likewise, if $\beta^{\prime }=\left\{\alpha_{1},\cdots ,\beta_{K},\beta_{K+1}\right\}$ with $\beta_{K}\subseteq \beta_{K+1}$ then $I_{\cap }^{\alpha \to \beta^{\prime }}=I_{\cap }^{\alpha \to \beta }$.

Intuitively, the first axiom guarantees that any double-redundancy function in ΦID reduces to a PID-type redundancy function when evaluated in certain atoms. The second encapsulates the basic desideratum that the double-redundancy does not change by adding sources and targets that are already considered, implying that one does not need to define them over combination of atoms beyond what is covered by the product lattice.

By exploiting these two axioms, one can define “atoms” that belong to each of the nodes via the Moebius inversion formula. Concretely, the ΦID *atoms*$I_{\partial }^{\alpha \to \beta }$ are defined as the quantities that guarantee the following condition for all α→β∈A×A:

[9] $$\begin{array}{c} I_{\cap }^{\alpha \to \beta }=\sum_{\begin{array}{c} \alpha^{\prime }\to \beta^{\prime }⪯\alpha \to \beta \end{array}}I_{\partial }^{\alpha^{\prime }\to \beta^{\prime }}\;. \end{array}$$

In other words, $I_{\partial }^{\alpha \to \beta }$ corresponds to the information contained in node α→β and not in any node below it in the lattice. These are analogues to the redundant, unique, and synergistic atoms in standard PID, but using the product lattice as a scaffold. By inverting this relationship, one can find a recursive expression for calculating $I_{\partial }$ as

[10] $$\begin{array}{c} I_{\partial }^{\alpha \to \beta }=I_{\cap }^{\alpha \to \beta }-\sum_{\begin{array}{c} \alpha^{\prime }\to \beta^{\prime }≺\alpha \to \beta \end{array}}I_{\partial }^{\alpha^{\prime }\to \beta^{\prime }}. \end{array}$$

With all the tools at hand, we can deliver the promised decomposition of the TDMI in terms of atoms of integrated information, as established in the next definition.

Definition 1 The Integrated Information Decomposition (ΦID) of a system with Markovian dynamics is the collection of atoms $I_{\partial }$ defined from the redundancies $I_{\cap }$ via Eq. **10**, which satisfy

[11] $$\begin{array}{c} \text{TDMI}=I\left(X;Y\right)=\sum_{\alpha ,\beta \in A}I_{\partial }^{\alpha \to \beta }\;. \end{array}$$

In this way, the ΦID of two time series gives 16 atoms that correspond to the lattice shown in Fig. 1*C*, which are computed via a linear transformation over the 16 redundancies. Importantly, Axioms 1 and 2 allow us to compute all the $I_{\cap }$ terms once a single-target PID redundancy function Red(·) has been chosen with the sole exception of $I_{\cap }^{\left\{1\}\{2\}\to \{1\}\{2\right\}}$. (This can be verified directly by studying the coefficients of the linear system of equations that relate redundancies and atoms.) All this is summarized in the following result.

Proposition 1 **(15-for-free).** Axioms 1 and 2 provide unique values for the 16 atoms of the product lattice after one defines i) a single-target redundancy function Red(·), and ii) an expression for $I_{\partial }^{\left\{1\}\{2\}\to \{1\}\{2\right\}}$.

Therefore, in the same way as in PID the definition of Red(·) gives 3 other terms (unique and synergy) as side-product, *Proposition* 1 shows that in ΦID the addition of the double-redundancy function $I_{\partial }^{\left\{1\}\{2\}\to \{1\}\{2\right\}}$ gives the 15 other terms for free. In *SI Appendix*, we describe ΦID extensions of two common PID redundancy functions (Ince’s CCS (62) and Barrett’s MMI (63) measures), which we use for all numerical examples in this paper.

Note also that ΦID is formally calculated on a joint distribution $p(X_{t},X_{t+1})$ as observed on data. There are multiple ways one can obtain such distribution: Indeed, one could conceivably take the transition probabilities $p(X_{t+1}|X_{t})$ of a system of interest and consider any input distribution $p(X_{t})$, and use the basic rules of probability to obtain $p(X_{t},X_{t+1})$. One could also choose an arbitrary $p(X_{t+1})$ and use Bayes rule to obtain the joint. One important caveat, naturally, is that one cannot choose any arbitrary $p(X_{t})$ *and* $p(X_{t+1})$, since they need to be consistent with the system’s dynamics. Here, our approach is to use the stationary distribution $p(X_{t},X_{t+1})$ of the system, which by construction is consistent with the system’s dynamics.

### Library of 1,053 Multivariate Time-Series Systems.

ref. 36 assembled a curated library of 1,053 multivariate time-series, including both real-world systems and synthetic ones, openly available at: https://doi.org/10.5281/zenodo.7118947. Each dataset comprises between 5 and 40 time-series, each between 100 and 2,000 time-points in length. A summary is provided in *SI Appendix*.

## Supplementary Material

Appendix 01 (PDF)

## Data Availability

Previously published data were used for this work (36, 76).

## Footnotes

^*^In non-Markovian systems the corresponding quantity is known as *excess entropy* (5). Information decomposition in non-Markovian systems will be covered in a subsequent publication.

^†^The relation between ΦID and causal emergence (45) can be found in refs. 46 and 47.

^‡^The parts typically being chosen so as to have the weakest informational link between them.

^§^The only possible partition of a 2-component system is its minimum-information partition.

^¶^We provide definitions of each measure in *SI Appendix*—for details, see section 2.2 of ref. 12 and the original refs. 7, 16, and 54.

^#^Or linear variants of it, to which our conclusions also apply.

^‖^Similar concerns about TE have been raised in ref. 43.

^**^The analysis focuses on pairs of regions only for convenience, as the theory applies to systems of any size.

^††^If PID told us that all the TE is due to synergistic effects, then we can also infer that any correlation with AIS is not due to information duplication. However, if a correlation between AIS and TE were present and any nonsynergistic TE were present, then even PID would not be enough to disentangle the contribution of ${\text{Un}}^{1}\to \text{Red}$ to any observed AIS-TE correlation.

^‡‡^For example, measures that accurately discriminate between neural configurations corresponding to conscious and unconscious states in a particular experimental paradigm (78).

^§§^For a case of N variables, then A is the set of antichains on the lattice (P({1,...,N}),⊆), discussed in ref. 17. We focus on the bivariate case for clarity, although the ΦID formalism developed here can be applied to any N.

^¶¶^A proof of this is provided in *SI Appendix*.

^##^Here, we use the shorthand notation $X^{\alpha }:=\left(X_{i_{1}},\cdots ,X_{i_{K}}\right)$ for $\alpha =\{i_{1},\cdots ,i_{K}\}$.
