# Supplementary material for: Toward a unified taxonomy of information dynamics via Integrated Information Decomposition
Source: Proc Natl Acad Sci U S A. 2025 Sep 22;122(39):e2423297122. doi: 10.1073/pnas.2423297122 (PMC12501198; doi:10.1073/pnas.2423297122)
Supplement: Supplementary file 1 — Appendix 01 (PDF) [file pnas.2423297122.sapp.pdf]

# Supporting Information for

# Towards a unified taxonomy of information dynamics via Integrated Information Decomposition

Pedro A.M. Mediano,[1,2,*] Fernando E. Rosas,[3,4,5,6,*] Andrea I. Luppi,[5,7,8]
Robin L. Carhart-Harris,[4,9] Daniel Bor,[10,11] Anil K. Seth,[3,12] and Adam B. Barrett[3,13]
[1] *Department of Computing, Imperial College London, London, UK*
[2] *Division of Psychology and Language Sciences, University College London, London, UK*
[3] *Sussex AI and Sussex Centre for Consciousness Science, Department of Informatics, University of Sussex*
[4] *Centre for Complexity Science and Center for Psychedelic Research, Imperial College London*
[5] *Centre for Eudaimonia and Human Flourishing, Department of Psychiatry, University of Oxford*
[6] *Principles of Intelligent Behavior in Biological and Social Systems (PIBBSS)*
[7] *St John's College, University of Cambridge, Cambridge, UK*
[8] *Division of Information Engineering, University of Cambridge*
[9] *Department of Neurology, University of California San Francisco*
[10] *Department of Psychology, Queen Mary University of London*
[11] *Department of Psychology, University of Cambridge, Cambridge, UK*
[12] *Program on Brain, Mind, and Consciousness, Canadian Institute for Advanced Research (CIFAR)*
[13] *The Data Intensive Science Centre, University of Sussex, Brighton, UK*
(Dated: August 19, 2025)

## I. THE PRODUCT OF TWO LATTICES IS A LATTICE

A lattice is a partially ordered set $(\mathcal{A}, \preceq)$ for which every pair of elements $a, b$ has a well-defined *meet* $a \wedge b$ and *join* $a \vee b$, which correspond to their common greatest lower bound (infimum) and common least upper bound (supremum), respectively [1]. Here we prove that, if $(\mathcal{A}, \preceq)$ is a lattice, then the product lattice $(\mathcal{A} \times \mathcal{A}, \preceq^*)$ equipped with the order relationship

$$\alpha \to \beta \preceq^* \alpha' \to \beta' \quad \text{if and only if} \quad \alpha \preceq \alpha' \text{ and } \beta \preceq \beta', \tag{1}$$

is also a lattice, where $\alpha, \beta, \alpha', \beta' \in \mathcal{A}$. As a corollary of this, given that the set and partial ordering relationship used in PID are a lattice [2, 3], then the set and partial ordering relationship used in $\Phi$ID are also a lattice.

For compactness, let us use the notation $\gamma = \alpha \to \beta$ and $\gamma' = \alpha' \to \beta'$ for $\gamma, \gamma' \in \mathcal{A} \times \mathcal{A}$. To prove the lattice structure of $(\mathcal{A} \times \mathcal{A}, \preceq^*)$ it suffices to show that

1. $\gamma \wedge^* \gamma' := \alpha \wedge \alpha' \to \beta \wedge \beta'$ is a valid meet; and
2. $\gamma \vee^* \gamma' := \alpha \vee \alpha' \to \beta \vee \beta'$ is a valid join.

Note that the fact that $(\mathcal{A}, \preceq)$ is a lattice implies that $\alpha \wedge \beta$ and $\alpha \vee \beta$ are well-defined for all $\alpha, \beta \in \mathcal{A}$.

Let us begin with the meet, for which we use $m = \gamma \wedge^* \gamma'$ as a shorthand notation. First, one can directly check that $m \preceq^* \gamma$ and $m \preceq^* \gamma'$, given the definition of $\preceq^*$ above and the fact that $\alpha \wedge \alpha' \preceq \alpha$ (and similarly for $\alpha'$, $\beta$, and $\beta'$). Next, we need to prove that for any $\gamma'' = \alpha'' \to \beta'' \in \mathcal{A} \times \mathcal{A}$ such that $\gamma'' \preceq^* \gamma$ and $\gamma'' \preceq^* \gamma'$, we have $\gamma'' \preceq^* m$ (i.e. that $m$ is the greatest lower bound of $\gamma$ and $\gamma'$). To see this, note that the conditions $\gamma'' \preceq^* \gamma$ and $\gamma'' \preceq^* \gamma'$ imply the following four statements:

$$\begin{aligned} \alpha'' &\preceq \alpha \ , \\ \alpha'' &\preceq \alpha' \ , \\ \beta'' &\preceq \beta \ , \\ \beta'' &\preceq \beta' \ . \end{aligned}$$

---

* P.M. and F.R. contributed equally to this work.
E-mail: p.mediano@imperial.ac.uk; f.rosas@sussex.ac.uk

Using these relationships and the $\wedge$ operator from $\mathcal{A}$, one can show that $\alpha'' \preceq \alpha \wedge \alpha'$ and $\beta'' \preceq \beta \wedge \beta'$, which in turn implies that $\gamma'' \preceq^* m$. Finally, the proof for the join is analogous, replacing $\wedge$ with $\vee$ and $\preceq$ with $\succeq$.

## II. DECOMPOSING PID ATOMS

Equation (4) in the main text shows how to decompose redundancies in the product lattice in terms of ΦID atoms. Here we provide a more general statement, that allows us to decompose not only redundancies, but also other PID atoms. The goal of this appendix is to build stronger connections between PID and ΦID, and to extend Proposition 1 to allow greater flexibility for specifying a ΦID function.

Note that the ΦID framework applies to any pair of sets of source and target variables, which need not correspond to the past and future states of a dynamical system. To highlight the generality of the ΦID framework, for the rest of this supplementary material we use the notation $\boldsymbol{X} = \{X_1, X_2, ...\}$ to refer to the sources, and analogously $\boldsymbol{Y} = \{Y_1, Y_2, ...\}$ for the targets. The expressions in the main text can be recovered by simply setting $\boldsymbol{X} \coloneqq \boldsymbol{X}_t, \boldsymbol{Y} \coloneqq \boldsymbol{X}_{t+1}$.

For the forward PID, and borrowing the notation from Williams and Beer [2], given a non-empty set of 'future' variables $F \in \mathcal{P}(\{Y_1, ..., Y_N\})$ and an an element of the redundancy lattice $\boldsymbol{\alpha} \in \mathcal{A}$, let us denote by $\Pi_F(\boldsymbol{\alpha}; F)$ the $\boldsymbol{\alpha}$ atom of the PID decomposition for $I(\boldsymbol{X}; F)$, such that

$$I(\boldsymbol{X}; F) = \sum_{\boldsymbol{\alpha} \in \mathcal{A}} \Pi_F(\boldsymbol{\alpha}; F) \ . \tag{2}$$

We use an analogous notation for the backward PID, with a corresponding non-empty set of 'past' variables $P \in \mathcal{P}(\{X_1, ..., X_N\})$ and $\boldsymbol{\beta} \in \mathcal{A}$, such that

$$I(P; \boldsymbol{Y}) = \sum_{\boldsymbol{\beta} \in \mathcal{A}} \Pi_B(P; \boldsymbol{\beta}) \ . \tag{3}$$

Then, these quantities can be further decomposed in ΦID atoms as

$$\Pi_F(\boldsymbol{\alpha}; F) = \sum_{\gamma \preceq F} I_\partial^{\boldsymbol{\alpha} \to \gamma} \ , \tag{4a}$$

$$\Pi_B(P; \boldsymbol{\beta}) = \sum_{\gamma \preceq P} I_\partial^{\gamma \to \boldsymbol{\beta}} \ . \tag{4b}$$

Note that the sum runs only across one of the sets (instead of both as it does in Eq. (4) of the main text), and that every element in $\mathcal{P}(\{1, ..., N\})$ is also in $\mathcal{A}$, and hence the partial order relationship in the sums above is well-defined. As a few examples, in a bivariate system the following forward PID atoms decompose as:

$$\begin{aligned}
\texttt{Red}(X_1, X_2; Y_i) &= \Pi_F(\{1\}\{2\}; Y_i) \\
&= I_\partial^{\{1\}\{2\} \to \{1\}\{2\}} + I_\partial^{\{1\}\{2\} \to \{i\}} \ , \\
\\
\texttt{Syn}(X_1, X_2; Y_i) &= \Pi_F(\{12\}; Y_i) \\
&= I_\partial^{\{12\} \to \{1\}\{2\}} + I_\partial^{\{12\} \to \{i\}} \ , \\
\\
\texttt{Un}(X_1; Y_1 Y_2 | X_2) &= \Pi_F(\{1\}; Y_1 Y_2) \\
&= I_\partial^{\{1\} \to \{1\}\{2\}} + I_\partial^{\{1\} \to \{1\}} \\
&\quad + I_\partial^{\{1\} \to \{2\}} + I_\partial^{\{1\} \to \{12\}} \ .
\end{aligned}$$

These decompositions can be used to prove Proposition 1 of the main text. Adopting a view of ΦID as a linear system of equations, one needs 16 independent equations to solve for the 16 unknowns that are the ΦID atoms. Of those, 9 are given by standard Shannon mutual information (specifically, $I(X_i; Y_j)$, $I(X_1 X_2; Y_i)$, $I(Y_1 Y_2; X_i)$, and $I(X_1 X_2; Y_1 Y_2)$, for $i, j = \{1, 2\}$) decomposed with Eq. (4) of the main text, and 6 are given by the single-target PIDs ($\texttt{Red}(X_1, X_2; Y_1)$, $\texttt{Red}(X_1, X_2; Y_2)$, and $\texttt{Red}(X_1, X_2; Y_1 Y_2)$, as well as the 3 corresponding backward PIDs) decomposed by the expression above. Finally, one only need to add one individual ΦID atom to make the 16 equations needed, and the system can be solved for all other atoms.

Taking these results together, Proposition 1 in the main text can be generalised as follows: a valid ΦID can be defined not only in terms of redundancy, but also in terms of unique information or synergy. This is equivalent to the case of PID, for which decompositions based on unique information [4] or synergy [5, 6] have been proposed.

## III. COMPUTING THE ΦID ATOMS

Computation of ΦID proceeds following the same general steps as in PID: first the intersection information $I_\cap^{\boldsymbol{\alpha}\to\boldsymbol{\beta}}$ is computed for every node; and then the integrated information atoms $I_\partial^{\boldsymbol{\alpha}\to\boldsymbol{\beta}}$ are obtained as the solution to a linear system of equations representing the Moebius inversion. Thus, the only element we need to specify is a double-redundancy function. We provide details of two double-redundancy functions, based on well-known PID redundancy functions [7, 8].

### A. Minimum mutual information

To compute the double-redundancy atom, $I_\partial^{\{1\}\{2\}\to\{1\}\{2\}}$, for numerical applications, we assume all systems are distributed as a multivariate Gaussian distribution, and use a ΦID extension of Barrett's Minimum Mutual Information (MMI) PID [7]:

**Definition 1.** ***Double-redundancy based on minimum mutual information****. For a given set of variables $(\boldsymbol{X}, \boldsymbol{Y})$, the double-redundancy based on minimum mutual information is defined as*

$$I_{\partial,\mathrm{MMI}}^{\{1\}\{2\}\to\{1\}\{2\}} := \min_{i,j} I(X_i; Y_j) \ . \tag{5}$$

The MMI PID quantifies redundancy in terms of the minimum mutual information of each individual source with the target; synergy, then, becomes identified with the additional information provided by the weaker source once the stronger source is known [7]. Crucially, this has been shown to be the most 'conservative' way of defining redundancy, in the sense that it is an upper bound to other possible definitions [7]. An attractive feature of MMI is that makes the redundancy only depend on the marginal distributions between individual sources and target, which has been highlighted as a desirable property in the literature [9]. However, a limitation of MMI is that it calculates redundancy based solely on the amount of information that each variable has about the target, which does not imply necessarily that this information comprises the same content [8]. Additionally, a peculiarity of MMI is its stringent definition of unique information, which is always zero for one of the two variables under the MMI definition of redundancy. By identifying redundancy with the minimum of the MIs between each source and the target, the source whose MI is the minimum will have redundancy equal to its MI with the target. Since a source's unique and redundant information must sum up to its MI with the target, this means that this source's unique information will be zero. In other words, the liberal definition of redundancy under MMI is at the expense of a stringent interpretation of unique information.

In PID, MMI is a totally monotonic function on the redundancy lattice, and therefore yields a non-negative decomposition. It is worth noting that this does not hold in ΦID – the MMI double-redundancy is monotonic (but not totally monotonic) on the double-redundancy lattice, and thus can lead to negative ΦID atoms.

All results in the main text were computed with the MMI ΦID, and for completeness Sec. IX of this Appendix shows all results replicated with an alternative ΦID based on common change in surprisal, described below.

### B. Common change in surprisal

To show the presented results do not depend on the specific choice of the MMI function, we also formulate a ΦID extension of Ince's *Common Change in Surprisal* (CCS) redundancy function [8]. As per the compatibility axiom, we formulate a multi-target CCS function that reduces to the original when only a single target is specified.

In line with Ince [8], we define $I_{\partial,\mathrm{CCS}}^{\{1\}\{2\}\to\{1\}\{2\}}$ using pointwise (or *local*) information measures [10]. As a first step, we use the inclusion-exclusion principle to formulate a local 'multi-target co-information' $c(\boldsymbol{x};\boldsymbol{y})$, defined as

$$c(\boldsymbol{x};\boldsymbol{y}) := \sum_{\boldsymbol{\alpha}\to\boldsymbol{\beta}\in\overline{\mathcal{A}^2}} (-1)^{f(\boldsymbol{\alpha},\boldsymbol{\beta})+1} \; i_\cap^{\boldsymbol{\alpha}\to\boldsymbol{\beta}}(\boldsymbol{x};\boldsymbol{y}) \ , \tag{6}$$

where $i_\cap^{\boldsymbol{\alpha}\to\boldsymbol{\beta}}(\boldsymbol{x};\boldsymbol{y})$ is the pointwise redundancy function, $\overline{\mathcal{A}^2}$ is the set of nodes in the product lattice excluding the lowest node, and $f(\boldsymbol{\alpha},\boldsymbol{\beta}) = \sum_{a\in\boldsymbol{\alpha}} |a| + \sum_{b\in\boldsymbol{\beta}} |b|$ [11]. Note that $i_\cap^{\boldsymbol{\alpha}\to\boldsymbol{\beta}}(\boldsymbol{x};\boldsymbol{y})$ above corresponds to the standard pointwise mutual information and a single-target PID redundancy function, which we take here to be the usual CCS function as defined by Ince [8]. For the bivariate ΦID, a formal calculation shows that

$$c(\boldsymbol{x};\boldsymbol{y}) = i_\partial^{\{1\}\{2\}\to\{1\}\{2\}}(\boldsymbol{x};\boldsymbol{y}) - i_\partial^{\{12\}\to\{12\}}(\boldsymbol{x};\boldsymbol{y}) \ .$$

Please note that, as for the co-information in the standard PID case, $c(\boldsymbol{x};\boldsymbol{y})$ is a 'whole-minus-sum' measure[12] that can be computed without any ΦID atoms explicitly. Then, as the second step in the definition, given a large set of $M$ samples $\{\boldsymbol{x}^{(i)}, \boldsymbol{y}^{(i)}\}_{i=1}^{M}$ we define the set $\mathcal{S} \subseteq \{1, ..., M\}$ as the subset of samples for which all marginal pointwise mutual informations, as well as the pointwise full mutual information $I(\boldsymbol{X};\boldsymbol{Y})$, have the same sign. With this, we are finally able to define the Gaussian CCS double-redundancy function.

**Definition 2.** ***Double-redundancy based on common change in surprisal****. For a given set of variables $(\boldsymbol{X}, \boldsymbol{Y})$, the double-redundancy based on common change in surprisal is defined as*

$$I_{\partial,\mathrm{CCS}}^{\{1\}\{2\}\to\{1\}\{2\}} := \sum_{i\in\mathcal{S}} c(\boldsymbol{x}^{(i)};\boldsymbol{y}^{(i)}) \tag{7}$$

Note that in this definition, the CCS redundancy is calculated with respect to the distribution of the system of interest, $p(\boldsymbol{x},\boldsymbol{y})$, which corresponds to the definition in the original pre-print of Ref. [8]. In the subsequent published version, Ref. [8] calculates CCS with respect to a maximum entropy projection of $p$ under suitable constraints. An extension of this maximum entropy constraints to the ΦID case would be non-trivial, and therefore the numerical results in Sec. IX below use CCS without the maximum entropy projection, as per the original CCS definition. Developing a ΦID extension of CCS with a suitably defined maximum entropy projection is a promising avenue for future work.

In both cases (CCS and MMI), it is direct to check the proposed definitions satisfy Axiom 1 (compatibility) with respect to their original PID definitions [7, 8]. Additionally, it can be shown that both MMI and CCS satisfy Axiom 2 (subset equality): for MMI this is a direct property of the data processing inequality, while for CCS this has been proven in [8, Sec. 4.4]. That being said, it is worth keeping in mind that while the extensions presented here seem the most natural, they are not the only possible ones that are compatible with the originals, and in principle any function that satisfies the double-redundancy axioms can be used to compute ΦID.

## IV. RESULTS OF SECTION 'DIFFERENT TYPES OF INTEGRATION'

Here we present calculations for the example systems in Fig. 4 of the main text. These proofs hold for all ΦID that satisfy the partial ordering axiom of $I_\cap^{\boldsymbol{\alpha}\to\boldsymbol{\beta}}$ (Axiom 2 in the main text), have a non-negative double-redundancy function $I^{\{1\}\{2\}\to\{1\}\{2\}} \geq 0$, and satisfy the following bound that follows from the basic properties of PID (c.f. [13]):

$$\mathtt{Red}(X,Y;Z) \leq \min\{I(X;Z), I(Y;Z)\} \ . \tag{8}$$

Note that the MMI satisfies these properties (and thus we report the results in the main text), although CCS doesn't. Let us examine the three systems in turn:

- For the copy transfer system, $Y_2 = X_1$, while $X_2$ and $Y_2$ are independent i.i.d. fair coin flips. Since $Y_2$ is independent from the rest of the system, $\mathtt{Red}(X_1, X_2; Y_2) = \mathtt{Red}(X_1, X_2; Y_2) = 0$, and due to partial ordering $I_\cap^{\{1\}\{2\}\to\{1\}\{2\}} = 0$. Finally, using the Moebius inversion formula it follows that $I_\partial^{\{1\}\to\{2\}} = I(X_1;Y_2) = 1$ and all other atoms are zero.

- In the downward XOR system, $X_1$ and $X_2$ are i.i.d. fair coin flips, $Y_1 = X_1 \oplus X_2$, and $Y_2$ is independent of the rest. Then, it is clear that $I(X_1, X_2; Y_1, Y_2) = I(X_1, X_2; Y_1) = 1$, while $I(X_1; Y_1) = I(X_2; Y_1) = 0$. Additionally, note that $I_\cap^{\{12\}\to\{1\}\{2\}} = 0$, since $\mathtt{Red}(Y_1, Y_2; X_1X_2) \leq I(Y_2; X_1X_2) = 0$. All this implies that all the redundancies (and hence all the atoms) below $\{12\} \to \{1\}$ are zero, and hence $I_\partial^{\{12\}\to\{1\}} = 1$ due to the Moebius inversion formula.

- Finally, consider the PPR system where $X_1, X_2, Y_1$ are i.i.d. fair coin flips and $Y_2$ is such that $X_1 \oplus X_2 = Y_1 \oplus Y_2$. Then $I(X_1, X_2; Y_1) = I(X_1, X_2; Y_2) = I(X_1; Y_1, Y_2) = I(X_2; Y_1, Y_2) = 0$. This implies that all redundancies (and hence atoms) except $I_\cap^{\{12\}\to\{12\}}$ are zero, and hence using again the Moebius inversion formula $I_\partial^{\{12\}\to\{12\}} = I(X_1, X_2; Y_1, Y_2) = 1$.

## V. RESULTS RELATED TO MEASURES OF INTEGRATED INFORMATION

In this appendix we prove the results in Table 1 of the main text, that shows whether each of four measures of integrated information ($\Phi^{\mathrm{WMS}}$, CD, $\psi$, $\Phi_G$) are positive, negative, or zero in a system containing only one ΦID atom.

A succinct definition of each measure is given below, and a comprehensive review and comparison of these and other measures can be found in Ref. [14].

Throughout this section we focus on bivariate systems, and use $i, j$ as variable indices, with $i \neq j$. To complete the proof we will first show that it is possible to build systems with exactly one bit of information in one ΦID atom, and we will then compute the four measures on those systems.

Let us begin with the design of systems with one specific ΦID atom. Intuitively, this can be accomplished with a suitable combination of COPY and XOR gates for redundant and synergistic sets of variables, respectively. More formally, the procedure to build a system with $I_\partial^{\boldsymbol{\alpha}\to\boldsymbol{\beta}} = 1$ and all other atoms equal to zero is as follows:

1. Sample $w$ from a Bernoulli distribution with $p = 0.5$.
2. Sample $\boldsymbol{x}$ based on $\boldsymbol{\alpha}$:
   - If $\boldsymbol{\alpha} = \{1\}\{2\}$, then $x_1 = x_2 = w$.
   - If $\boldsymbol{\alpha} = \{i\}$, then $x_i = w$ and $x_j$ is sampled from a Bernoulli distribution with $p = 0.5$.
   - If $\boldsymbol{\alpha} = \{12\}$, then $\boldsymbol{x}$ is a random string with parity $w$.
3. Sample $\boldsymbol{y}$ based on $\boldsymbol{\beta}$ analogously.

In all cases there will be one bit of information ($w$) shared between $\boldsymbol{X}$ and $\boldsymbol{Y}$, hence $I(\boldsymbol{X};\boldsymbol{Y}) = 1$ for any choice of $\boldsymbol{\alpha}, \boldsymbol{\beta}$. This can be proven using the fact that for any $\boldsymbol{\alpha}, \boldsymbol{\beta}$, one has $H(W) = 1$, $H(W|\boldsymbol{X}) = H(W|\boldsymbol{Y}) = 0$, and $p(\boldsymbol{x}, \boldsymbol{y}, w) = p(\boldsymbol{x}|w)p(\boldsymbol{y}|w)p(w)$. To do so, let us start from the mutual information chain rule:

$$\begin{aligned} I(\boldsymbol{X};\boldsymbol{Y}W) &= I(\boldsymbol{X};W) + I(\boldsymbol{X};\boldsymbol{Y}|W) \\ &= I(\boldsymbol{X};\boldsymbol{Y}) + I(\boldsymbol{X};W|\boldsymbol{Y}) \ . \end{aligned}$$

Rearranging the above terms, one can find that

$$I(\boldsymbol{X};\boldsymbol{Y}) = I(\boldsymbol{X};W) + I(\boldsymbol{X};\boldsymbol{Y}|W) - I(\boldsymbol{X};W|\boldsymbol{Y}) \ ,$$

where $I(\boldsymbol{X};W) = H(W) - H(W|\boldsymbol{X}) = 1$ and $I(\boldsymbol{X};\boldsymbol{Y}|W) = 0$. Finally, one finds that

$$\begin{aligned} I(\boldsymbol{X};W|\boldsymbol{Y}) &= H(\boldsymbol{X}|\boldsymbol{Y}) + H(W|\boldsymbol{Y}) - H(\boldsymbol{X}W|\boldsymbol{Y}) \\ &= H(\boldsymbol{X}|\boldsymbol{Y}) + H(W|\boldsymbol{Y}) - \big[H(\boldsymbol{X}|\boldsymbol{Y}) + H(W|\boldsymbol{X}\boldsymbol{Y})\big] \\ &= 0 \ , \end{aligned}$$

which concludes the proof that $I(\boldsymbol{X};\boldsymbol{Y}) = 1$. Furthermore, following a procedure similar to those in the previous section, it can be shown that any ΦID that satisfies the axioms described above (partial ordering, non-negative double-redundancy, and upper-bounded redundancy) correctly assigns 1 bit of information to $I_\partial^{\boldsymbol{\alpha}\to\boldsymbol{\beta}}$, and 0 to all other atoms.

Now that we have built these 16 single-atom systems, let us move to the integration measures of interest. For CD, $\psi$, and $\Phi^{\mathrm{WMS}}$, we will proceed by decomposing them in terms of ΦID atoms and checking whether each atom is positive (+), negative (−), or absent (0) from the decomposition to obtain the results in Table 1 of the article. Let us begin with CD, defined as the sum of transfer entropies from one variable to the other:

$$\begin{aligned} \mathrm{CD} &= \frac{1}{2}\sum_{i=1}^{2} I(X_i;Y_j|X_j) \\ &= \frac{1}{2}\sum_{i=1}^{2}\left(I_\partial^{\{i\}\to\{1\}\{2\}} + I_\partial^{\{i\}\to\{j\}} + I_\partial^{\{12\}\to\{1\}\{2\}} + I_\partial^{\{12\}\to\{j\}}\right) \ . \end{aligned} \tag{9}$$

Similarly, for $\psi$ the atoms can be extracted from the decomposition of $\texttt{Syn}(X_1, X_2; Y_1Y_2)$ in Eq. (4a):

$$\begin{aligned} \psi &= \texttt{Syn}(X_1, X_2; Y_1Y_2) \\ &= I_\partial^{\{12\}\to\{1\}\{2\}} + I_\partial^{\{12\}\to\{1\}} + I_\partial^{\{12\}\to\{2\}} + I_\partial^{\{12\}\to\{12\}} \ . \end{aligned} \tag{10}$$

For $\Phi^{\mathrm{WMS}}$, the atoms can be extracted from the decomposition of Eq. (9) in the main text:

$$\Phi^{\mathrm{WMS}} = -\, I_\partial^{\{1\}\{2\}\to\{1\}\{2\}} + I_\partial^{\{1\}\{2\}\to\{12\}} + \psi + \sum_{i=1}^{2}\left(I_\partial^{\{i\}\to\{j\}} + I_\partial^{\{i\}\to\{12\}}\right) \ . \tag{11}$$

The $\Phi_G$ case is slightly more involved, since it is not easily decomposable into a sum of ΦID atoms. According to the definition of $\Phi_G$ [15], for a system given by the joint probability distribution $p(\boldsymbol{X}, \boldsymbol{Y})$ one has

$$\Phi_G = \min_{q \in \mathcal{M}_G} D_{\text{KL}}(p \| q) ,$$

where $\mathcal{M}_G$ is the manifold of probability distributions that satisfy the constraints

$$q(Y_i|\boldsymbol{X}) = q(Y_i|X_i) . \tag{12}$$

Therefore, it suffices to check whether the probability distribution of the system satisfies the constraints in Eq. (12) — if it does, then $\Phi_G = 0$, and otherwise $\Phi_G > 0$ — which can be easily verified for each system separately to obtain the $\Phi_G$ column in Table 1, concluding the proof.

## VI. RESULTS OF SECTION 'WHY WHOLE-MINUS-SUM Φ CAN BE NEGATIVE'

In this appendix we describe the details of the noisy autoregressive system and how to compute its ΦID to yield the results shown in Figure 5 of the main text.

Given the past state of the system $x^1_t, x^2_t$, the next state is given by

$$\begin{aligned} x^1_{t+1} &= a(x^1_t + x^2_t) + \varepsilon^1_{t+1} \\ x^2_{t+1} &= a(x^1_t + x^2_t) + \varepsilon^2_{t+1} , \end{aligned}$$

where $a = 0.4$ is a fixed coupling parameter and $\varepsilon^1_t, \varepsilon^2_t$ are zero-mean unit-variance white noise processes with correlation $c$. All information-theoretic functionals are computed with respect to the system's stationary distribution, which can be shown to be a Gaussian and analytically calculated by means of a discrete Lyapunov equation [14, 16]. Once this distribution is obtained, the values of the atoms can be obtained following the procedures in Sec. III above.

## VII. FANTASIA DATASET

The Fantasia database [17] is an openly available dataset that contains data from 40 healthy subjects who were monitored via time-synchronized measurements of electrocardiogram (ECG) and respiration (impedance plethysmography) for two hours in supine resting position, while watching the Disney movie 'Fantasia.' All signals were recorded at 250 Hz sampling frequency. Please see the original publication for further details [17], and Ref. [18] for details of the preprocessing pipeline.

## VIII. SIMULATION AND ANALYSIS OF WHOLE-BRAIN COMPUTATIONAL MODEL

To explore information decomposition in realistic neurophysiological data we study the Dynamic Mean-Field (DMF) model by Deco *et al.* [19, 20], which consists of a set of coupled differential equations modelling the average activity of multiple interacting brain regions. These equations represent each brain region as two reciprocally coupled neuronal populations, one excitatory and one inhibitory, with the corresponding synaptic currents $I^{(\text{E})}$ and $I^{(\text{I})}$ are mediated by NMDA and $\text{GABA}_\text{A}$ receptors respectively. Different brain regions are coupled via their excitatory populations only, and the structural connectivity is given by the matrix $C$. The structural connectivity matrix was obtained from the HCP 900 subjects data release [21, 22], and was preprocessed in the same was as in Ref. [23], resulting in an 83×83 connectivity matrix corresponding to the Lausanne-83 brain parcellation [24]. For all other aspects of model configuration and simulation we follow Herzog *et al.* [25], and reproduce all relevant details here for convenience.

TABLE S1. Dynamic Mean Field (DMF) model parameters

| *Parameter* | *Symbol* | *Value* |
|---|---|---|
| External current | $I_0$ | 0.382 nA |
| Excitatory scaling factor for $I_0$ | $W_{\mathrm{E}}$ | 1 |
| Inhibitory scaling factor for $I_0$ | $W_{\mathrm{I}}$ | 0.7 |
| Local excitatory recurrence | $w_+$ | 1.4 |
| Excitatory synaptic coupling | $J_{\mathrm{NMDA}}$ | 0.15 nA |
| Threshold for $F(I_n^{(\mathrm{E})})$ | $I_{\mathrm{thr}}^{(\mathrm{E})}$ | 0.403 nA |
| Threshold for $F(I_n^{(\mathrm{I})})$ | $I_{\mathrm{thr}}^{(\mathrm{I})}$ | 0.288 nA |
| Gain factor of $F(I_n^{(\mathrm{E})})$ | $g_{\mathrm{E}}$ | 310 nC$^{-1}$ |
| Gain factor of $F(I_n^{(\mathrm{I})})$ | $g_{\mathrm{I}}$ | 615 nC$^{-1}$ |
| Shape of $F(I_n^{(\mathrm{E})})$ around $I_{\mathrm{thr}}^{(\mathrm{E})}$ | $d_{\mathrm{E}}$ | 0.16 s |
| Shape of $F(I_n^{(\mathrm{I})})$ around $I_{\mathrm{thr}}^{(\mathrm{I})}$ | $d_{\mathrm{I}}$ | 0.087 s |
| Excitatory kinetic parameter | $\gamma$ | 0.641 |
| Amplitude of uncorrelated Gaussian noise $v_n$ | $\sigma$ | 0.01 nA |
| Time constant of NMDA | $\tau_{\mathrm{NMDA}}$ | 100 ms |
| Time constant of GABA | $\tau_{\mathrm{GABA}}$ | 10 ms |

The full model is given by

$$
\begin{aligned}
I_j^{(\mathrm{E})} &= W_{\mathrm{E}} I_0 + w_+ J_{\mathrm{NMDA}} S_j^{(\mathrm{E})} + G J_{\mathrm{NMDA}} \sum_{k=1}^{N} C_{jk} S_k^{(\mathrm{E})} - J_j^{\mathrm{FIC}} S_j^{(\mathrm{I})} \\
I_j^{(\mathrm{I})} &= W_{\mathrm{I}} I_0 + J_{\mathrm{NMDA}} S_j^{(\mathrm{E})} - S_j^{(\mathrm{I})} \\
r_j^{(\mathrm{E})} &= F\left(I_j^{(\mathrm{E})}\right) = \frac{g_{\mathrm{E}}\left(I_j^{(\mathrm{E})} - I_{\mathrm{thr}}^{(\mathrm{E})}\right)}{1 - \exp\left(-d_{\mathrm{E}}\, g_{\mathrm{E}}\left(I_j^{(\mathrm{E})} - I_{\mathrm{thr}}^{(\mathrm{E})}\right)\right)} \\
r_j^{(\mathrm{I})} &= F\left(I_j^{(\mathrm{I})}\right) = \frac{g_{\mathrm{I}}\left(I_j^{(\mathrm{I})} - I_{\mathrm{thr}}^{(\mathrm{I})}\right)}{1 - \exp\left(-d_{\mathrm{I}}\, g_{\mathrm{I}}\left(I_j^{(\mathrm{I})} - I_{\mathrm{thr}}^{(\mathrm{I})}\right)\right)} \\
\frac{dS_j^{(\mathrm{E})}(t)}{dt} &= -\frac{S_j^{(\mathrm{E})}}{\tau_{\mathrm{NMDA}}} + \left(1 - S_j^{(\mathrm{E})}\right) \gamma r_j^{(\mathrm{E})} + \sigma v_j(t) \\
\frac{dS_j^{(\mathrm{I})}(t)}{dt} &= -\frac{S_j^{(\mathrm{I})}}{\tau_{\mathrm{GABA}}} + r_j^{(\mathrm{I})} + \sigma v_j(t)
\end{aligned}
$$

Above, $j, k$ are indices that run across all $N$ brain regions; $F$ is the $F$-$I$ curve relating input current to output firing rate of a neural population; $J^{\mathrm{FIC}}$ is the feedback inhibitory control parameter, optimised to yield average firing rates of approximately 3 Hz; and the sub- and superscripts E/I denote excitatory/inhibitory quantities, respectively. The model was simulated using a standard Euler-Maruyama integration method [26], using the parameter values shown in Table S1. Note that all parameter values are fixed except the global coupling $G$, which we vary across simulations. Finally, the distributions of the simulated BOLD signals are approximated via Gaussian distributions, and the procedures in Sec. III above are applied to obtain the values of all ΦID atoms.

## IX. NUMERICAL RESULTS REPLICATED WITH ALTERNATIVE ΦID MEASURES

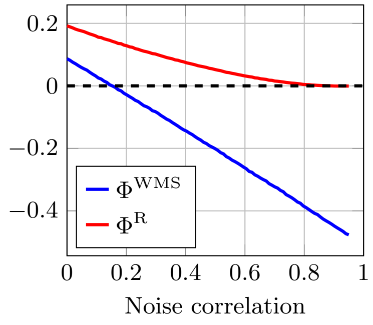


FIG. S1. **Results in two-node AR system replicated with CCS double-redundancy**.

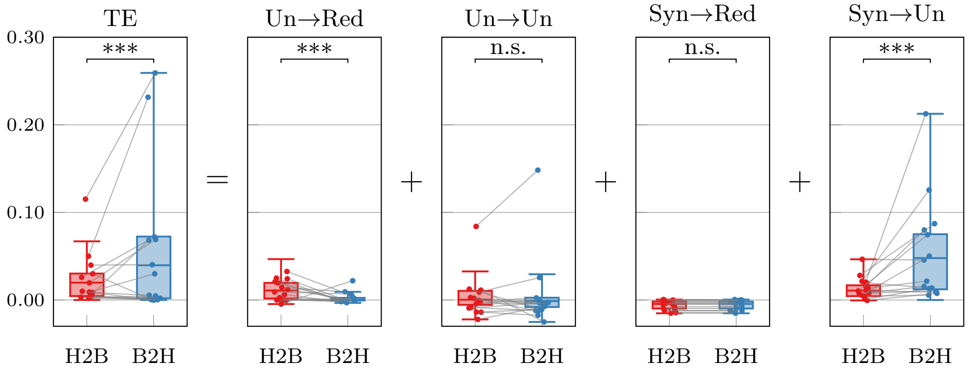


FIG. S2. **Results in Fantasia dataset replicated with CCS double-redundancy**.

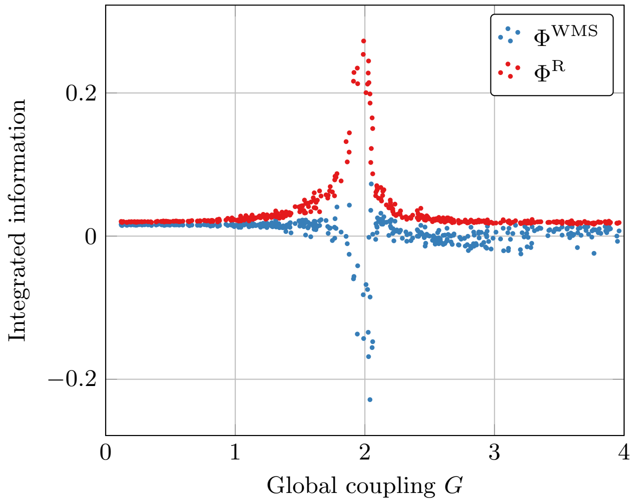


FIG. S3. **Results in DMF whole-brain simulation replicated with CCS double-redundancy**.

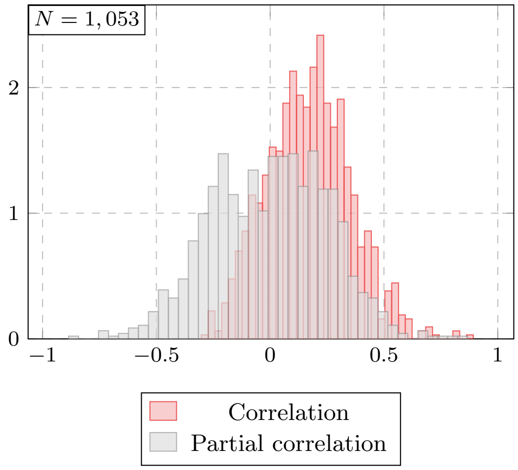


FIG. S4. **Results in the PYSPY dataset replicated with CCS double-redundancy**. Correlation between TE and AIS reduces (on average) from 0.17 to 0.002 after conditioning on $\texttt{Un}^1 \to \texttt{Red}$ ($t = 24.5$, $p < 10^{-5}$).

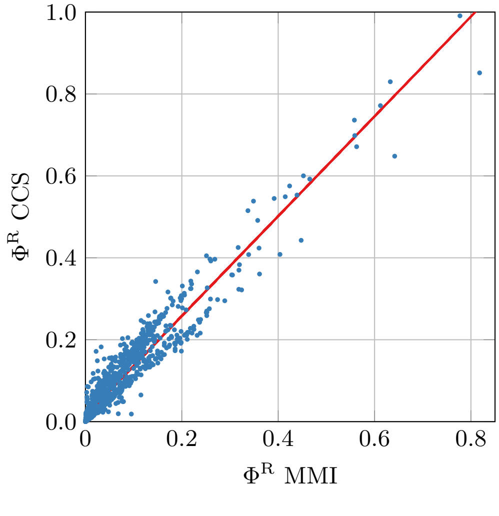


FIG. S5. **Integrated information $\Phi^{\mathrm{R}}$ is consistent between MMI and CCS across 1,053 different systems in the PYSPY dataset**. Spearman correlation between $\Phi^{\mathrm{R}}$ obtained with the MMI or CCS double-redundancy functions is 0.95 ($p < 0.001$).

[1] C. A. Charalambides, *Enumerative Combinatorics* (Chapman and Hall/CRC, 2002).
[2] P. L. Williams and R. D. Beer, Nonnegative decomposition of multivariate information (2010), arXiv:1004.2515 [cs.IT].
[3] J. Crampton and G. Loizou, The completion of a poset in a lattice of antichains, International Mathematical Journal **1**, 223 (2001).
[4] R. G. James, J. Emenheiser, and J. P. Crutchfield, Unique information via dependency constraints, Journal of Physics A: Mathematical and Theoretical **52**, 014002 (2018).
[5] R. Quax, O. Har-Shemesh, and P. Sloot, Quantifying synergistic information using intermediate stochastic variables, Entropy **19**, 85 (2017).
[6] F. E. Rosas, P. A. Mediano, B. Rassouli, and A. B. Barrett, An operational information decomposition via synergistic disclosure, Journal of Physics A: Mathematical and Theoretical **53**, 485001 (2020).
[7] A. B. Barrett, Exploration of synergistic and redundant information sharing in static and dynamical Gaussian systems, Phys. Rev. E **91**, 052802 (2015).
[8] R. A. A. Ince, Measuring multivariate redundant information with pointwise common change in surprisal, Entropy **19**, 10.3390/e19070318 (2017).
[9] N. Bertschinger, J. Rauh, E. Olbrich, J. Jost, and N. Ay, Quantifying unique information, Entropy **16**, 2161 (2014).
[10] For the rationale behind and extended discussion of local information measures see Lizier [27].
[11] Equivalently, $f(\boldsymbol{\alpha}, \boldsymbol{\beta})$ represents the length of the shortest path in the product lattice between the node $\boldsymbol{\alpha} \to \boldsymbol{\beta}$ and the lowest node.
[12] F. E. Rosas, P. A. Mediano, M. Gastpar, and H. J. Jensen, Quantifying high-order interdependencies via multivariate extensions of the mutual information, Physical Review E **100**, 032305 (2019).
[13] F. Rosas, V. Ntranos, C. Ellison, S. Pollin, and M. Verhelst, Understanding interdependency through complex information sharing, Entropy **18**, 38 (2016).
[14] P. Mediano, A. Seth, and A. Barrett, Measuring integrated information: Comparison of candidate measures in theory and simulation, Entropy **21**, 17 (2019).
[15] M. Oizumi, N. Tsuchiya, and S.-i. Amari, Unified framework for information integration based on information geometry, Proceedings of the National Academy of Sciences **113**, 14817 (2016).
[16] A. B. Barrett and A. K. Seth, Practical measures of integrated information for time-series data, PLoS Computational Biology **7**, 1 (2011).
[17] N. Iyengar, C. Peng, R. Morin, A. L. Goldberger, and L. A. Lipsitz, Age-related alterations in the fractal scaling of cardiac interbeat interval dynamics, American Journal of Physiology – Regulatory, Integrative and Comparative Physiology **271**, R1078 (1996).
[18] S. Nemati, B. A. Edwards, J. Lee, B. Pittman-Polletta, J. P. Butler, and A. Malhotra, Respiration and heart rate complexity: effects of age and gender assessed by band-limited transfer entropy, Respiratory Physiology & Neurobiology **189**, 27 (2013).
[19] G. Deco, A. Ponce-Alvarez, P. Hagmann, G. L. Romani, D. Mantini, and M. Corbetta, How local excitation-inhibition ratio impacts the whole brain dynamics, Journal of Neuroscience **34**, 7886 (2014).
[20] G. Deco, J. Cruzat, J. Cabral, G. M. Knudsen, R. L. Carhart-Harris, P. C. Whybrow, N. K. Logothetis, and M. L. Kringelbach, Whole-brain multimodal neuroimaging model using serotonin receptor maps explains non-linear functional effects of LSD, Current Biology **28**, 1 (2018).
[21] D. C. Van Essen, S. M. Smith, D. M. Barch, T. E. Behrens, E. Yacoub, K. Ugurbil, W.-M. H. Consortium, *et al.*, The WU-Minn Human Connectome Project: An overview, NeuroImage **80**, 62 (2013).
[22] M. F. Glasser, S. N. Sotiropoulos, J. A. Wilson, T. S. Coalson, B. Fischl, J. L. Andersson, J. Xu, S. Jbabdi, M. Webster, J. R. Polimeni, *et al.*, The minimal preprocessing pipelines for the Human Connectome Project, NeuroImage **80**, 105 (2013).
[23] A. I. Luppi and E. A. Stamatakis, Combining network topology and information theory to construct representative brain networks, Network Neuroscience **1**, 1 (2020).
[24] L. Cammoun, X. Gigandet, D. Meskaldji, J. P. Thiran, O. Sporns, K. Q. Do, P. Maeder, R. Meuli, and P. Hagmann, Mapping the human connectome at multiple scales with diffusion spectrum MRI, Journal of Neuroscience Methods **203**, 386 (2012).
[25] R. Herzog, P. A. Mediano, F. E. Rosas, R. Carhart-Harris, Y. Sanz, E. Tagliazucchi, and R. Cofré, A mechanistic model of the neural entropy increase elicited by psychedelic drugs, Scientific Reports **10** (2020).
[26] P. E. Kloeden and E. Platen, *Numerical Solution of Stochastic Differential Equations*, Vol. 23 (Springer, 2013).
[27] J. Lizier, *The Local Information Dynamics of Distributed Computation in Complex Systems* (University of Sydney, 2010).
